# Inflammatory neutrophil responses and T cell activation in ART-treated SIVmac239-infected rhesus macaques

**DOI:** 10.1093/jimmun/vkaf100

**Published:** 2025-06-12

**Authors:** Sallie L Fell, Sydney M Nemphos, James E Prusak, Hannah C Green, Jordyn Miller, Samuel Q Rowan, Natalie Valencia, Coty Tatum, Mary B Barnes, Carolina Allers, Sarah Scheuermann, Kelly Goff, Clara Krzykwa, Lori A Rowe, Nicholas J Maness, Matilda J Moström, Tiffany Hensley-McBain, Lara Doyle-Meyers, Amitinder Kaur, Jennifer A Manuzak

**Affiliations:** Division of Immunology, Tulane National Primate Research Center, Covington, LA, United States; Department of Microbiology and Immunology, Tulane University School of Medicine, New Orleans, LA, United States; Division of Immunology, Tulane National Primate Research Center, Covington, LA, United States; Department of Microbiology and Immunology, Tulane University School of Medicine, New Orleans, LA, United States; Division of Immunology, Tulane National Primate Research Center, Covington, LA, United States; Department of Microbiology and Immunology, Tulane University School of Medicine, New Orleans, LA, United States; Division of Immunology, Tulane National Primate Research Center, Covington, LA, United States; Division of Immunology, Tulane National Primate Research Center, Covington, LA, United States; Division of Immunology, Tulane National Primate Research Center, Covington, LA, United States; Division of Immunology, Tulane National Primate Research Center, Covington, LA, United States; Division of Microbiology, Tulane National Primate Research Center, Covington, LA, United States; Division of Microbiology, Tulane National Primate Research Center, Covington, LA, United States; Division of Immunology, Tulane National Primate Research Center, Covington, LA, United States; Division of Microbiology, Tulane National Primate Research Center, Covington, LA, United States; Division of Microbiology, Tulane National Primate Research Center, Covington, LA, United States; Division of Microbiology, Tulane National Primate Research Center, Covington, LA, United States; Division of Microbiology, Tulane National Primate Research Center, Covington, LA, United States; Division of Microbiology, Tulane National Primate Research Center, Covington, LA, United States; Department of Microbiology and Immunology, Tulane University School of Medicine, New Orleans, LA, United States; Division of Microbiology, Tulane National Primate Research Center, Covington, LA, United States; Division of Immunology, Tulane National Primate Research Center, Covington, LA, United States; Weissman Hood Institute, Great Falls, MT, United States; Division of Veterinary Medicine, Tulane National Primate Research Center, Covington, LA, United States; Division of Immunology, Tulane National Primate Research Center, Covington, LA, United States; Department of Microbiology and Immunology, Tulane University School of Medicine, New Orleans, LA, United States; Division of Immunology, Tulane National Primate Research Center, Covington, LA, United States; Department of Microbiology and Immunology, Tulane University School of Medicine, New Orleans, LA, United States

**Keywords:** antiretroviral therapy, immune activation, neutrophils, rhesus macaque, simian immunodeficiency virus

## Abstract

Modern antiretroviral therapy (ART) regimens have revolutionized the management of human immunodeficiency virus (HIV) and transformed it from a life-threatening disease to a manageable chronic condition. Despite the durable viral suppression associated with ART adherence, people with HIV (PWH) continue to experience chronic immune activation and inflammation, which has been linked with increased risk of developing non-acquired immunodeficiency syndrome (AIDS) comorbidities, including cardiovascular disease, liver disease, or neurocognitive disorders. Importantly, the mechanisms underlying establishment and maintenance of immune activation in ART-treated PWH remain incompletely defined. Here, we used a nonhuman primate model to evaluate associations between markers of systemic immune activation and peripheral neutrophils in simian immunodeficiency virus (SIV)-infected rhesus macaques (RMs), both before and after ART. As expected, peripheral frequencies of activated CD4^+^ and CD8^+^ T cells were elevated during acute SIV infection and returned to baseline levels following ART initiation. Neutrophil dynamics were impacted during acute SIV infection, including decreased peripheral neutrophil frequencies, increased neutrophil degranulation, and the potential for increased neutrophil extracellular trap (NET) formation. Treatment with ART mitigated these inflammatory neutrophil effector functions. Finally, frequencies of HLA-DR^+^ CD4^+^ and CD8^+^ T cells were significantly positively correlated with frequencies of inflammatory CD62L^dim^ neutrophils and plasma levels of myeloperoxidase, a component of neutrophil granules. Taken together, these data indicate that neutrophil activity and systemic T cell activation are correlated during acute SIV and early ART. Our work provides insight into associations between neutrophil dynamics and immune activation during HIV/SIV in the context of ART.

## Introduction

Despite significant advances in treatment and prevention options, human immunodeficiency virus (HIV) remains one of the world’s largest public health burdens. The World Health Organization (WHO) estimated that in 2023, there were 39.9 million people with HIV (PWH) and approximately 630,000 HIV-related deaths.^1^ Importantly, with consistent antiretroviral therapy (ART), PWH can suppress viral replication to non-detectable levels and experience improved health and quality of life. Current clinical recommendations suggest initiation of ART as soon as possible after diagnosis of HIV infection, which is associated with benefits including rapid virologic suppression, enhanced T cell recovery, reduced T cell activation, and smaller HIV reservoir size.^2–5^ However, treatment with ART does not constitute a cure for HIV and even with early ART initiation, PWH experience greater levels of inflammation and immune activation as compared to uninfected individuals.^6–9^

The precise mechanisms underlying persistent inflammation and immune activation in ART-treated PWH remain incompletely understood. Previous work has demonstrated that chronic inflammation in PWH may be mediated by residual HIV replication, gastrointestinal microbial dysbiosis, and microbial translocation, which results in continued activation of myeloid subsets including dendritic cells, monocytes, and macrophages.^10–12^ Neutrophils are a subset of myeloid-lineage innate immune cells which comprise up to 70% of all circulating leukocytes, leading them to be the most abundant white blood cell in the human immune system.^13^ Neutrophils are key players in the anti-viral response through effector functions such as degranulation and neutrophil extracellular trap (NET) formation.^14^ Importantly, prior work has shown that neutrophils exhibit both protective and detrimental responses during HIV infection.^15^ The protective role of neutrophils in anti-HIV responses involves release of myeloperoxidase (MPO) and reactive oxygen species, which are viricidal to human immunodeficiency virus type 1 (HIV-1) in vitro.^16^ Moreover, in vitro work has demonstrated that the anti-HIV properties of neutrophil-derived defensins and MPO can be concentrated and directed by capturing virions in NETs.^17^ Conversely, prior reports have demonstrated that chronic HIV infection results in neutrophil dysfunction, but the mechanism and timing by which this occurs remains unclear.^15^ A study in ART-treated children with HIV reported increased neutrophil oxidative burst during untreated infection and in the first 6 mo following ART initiation.^18^ Despite these strides in understanding the role of neutrophils in HIV, the contribution of other neutrophil effector functions, including degranulation and phagocytosis, to systemic immune activation remain to be elucidated.

The utilization of the nonhuman primate (NHP) model has allowed for many critical advances in the HIV/AIDS research field, such as the characterization of viral tropism, disease pathogenesis, and immune responses following infection with the highly similar simian immunodeficiency virus (SIV).^19–21^ In particular, infection of rhesus macaques (*Macaca mulatta*; RMs) with SIV induces disease pathology that replicates progressive HIV infection in humans, such as CD4^+^ T cell depletion, as well as high peak and chronic plasma viral load (VL).^22–24^ Treatment of SIV-infected RMs with combination ART consisting of tenofovir (TDF), emtricitabine (FTC), and dolutegravir (DTG) is effective in suppressing peripheral viral replication.^25^ Importantly, SIV infection of RMs results in immune cell activation and inflammation similar to what has been observed in PWH,^26–28^ and this systemic immune activation persists despite ART in RMs.^29^ A prior study in pig tail macaques (PTMs) demonstrated that NET formation contributes to pathogenesis in ART-treated, SIV-infected PTMs, supporting the potential pathogenic role of neutrophils in ART-treated SIV.^30^

Here, we used the RM model to examine T lymphocyte proliferation and activation, as well as function of peripheral neutrophils during acute SIV infection and following ART initiation. We hypothesized that inflammatory neutrophil responses would associate with elevated markers of systemic immune activation during acute SIV infection, which would persist despite initiation of daily ART. To test this hypothesis, we inoculated 6 adult, female RMs with SIVmac239, initiated daily ART 12 wk after SIV inoculation, and longitudinally monitored clinical and immune markers with an emphasis on peripheral neutrophil function and T cell activation.

## Materials and methods

### Study animals and approval

Six adult (5 to 16 yr old at time of enrollment), female, Indian-origin RMs were utilized in this study (Table 1). Animals were housed and cared for at the Tulane National Primate Research Center (TNPRC), under a protocol approved by the Institutional Animal Care and Use Committee (IACUC; Office of Laboratory Animal Welfare Assurance Number A4499-01). Animal housing, care, and procedures were performed in Association for Assessment and Accreditation of Laboratory Animal Care accredited facilities (AAALAC Number 000594), compliant with regulations put forth by the United States Department of Agriculture, including the Animal Welfare Act (9 CFR) and the Animal Care Policy Manual, with the guidelines established by the National Research Council in the Guide for the Care and Use of Laboratory Animals and the Weatherall Report. All animals were negative for MHC class I alleles associated with SIV control, including Mamu-A*01, Mamu-B*08, and Mamu-B*17.^31–33^ Animals were co-housed indoors under climate-controlled conditions and a 12-hour light/12-hour dark cycle and were monitored daily to ensure animal welfare. Any abnormalities were recorded and reported to a veterinarian. Water was available ad libitum, and animals were fed commercial monkey chow (Purina LabDiet; PMI Nutrition International, Richmond, Indiana, USA), supplemented with fruits, vegetables, and foraging treats as a part of the TNPRC environmental enrichment program. All procedures were performed under the direction of TNPRC veterinarians. Anesthesia was used in accordance with TNPRC standard operating procedures.

**Table 1.** Demographics of RMs enrolled in this study.

Animal ID	Sex	Age (y)	Weight (kg)	Origin	Mamu-A Haplotype 1	Mamu-A Haplotype 2	Mamu-B Haplotype 1	Mamu-B Haplotype 2
RM23-0171	F	11.75	10.85	Indian	A002.01	A016.01	B012.03	B024.01
RM23-0167	F	8.83	7.85	Indian	A002.01	–	B012.01	B024.01
RM23-0166	F	15.67	8.80	Indian	A002.01	A004.01	B002.01	B015.02
RM23-0172	F	8.83	6.50	Indian	A004.01	–	B002.01	B028.01
RM23-0169	F	4.83	6.40	Indian	A002.01	A049.01	B012.03	B069.01
RM23-0168	F	5.75	8.00	Indian	A004.01	A011.01	B048.01	–

Adult female rhesus macaques (RMs, n = 6) were enrolled in this study. Listed values of age and weight refer to the age and weight of animals 1 mo prior to being enrolled in the study. In Mamu haplotype columns, “-” indicates animal inherited same MHC haplotype from both parents.

### SIV inoculation, monitoring, and ART treatment

RMs were intravenously inoculated with 1 ml of 50 TCID50 SIVmac239 (SIVmac239 viral stock lot 5/12/15; in vitro titer: 1,811 TCID50/mL in CEMx174 cells) obtained from the TNPRC Virus Characterization, Isolation, Production and Sequencing Core. Plasma viral loads were monitored via reverse transcription quantitative polymerase chain reaction (RT-qPCR) by the TNPRC Pathogen Detection and Quantification Core, as previously described.^34^

At 12 wk post-infection, and continuing for the duration of the study, RMs were placed on a daily ART regimen, administered subcutaneously, consisting of tenofovir disoproxil fumarate (TDF; 5.1 mg/kg; Gilead, Foster City, California, USA), emtricitabine (FTC; 30 mg/kg; Gilead), and dolutegravir (DTG; 2.5 mg/kg; ViiV Healthcare, London, England, UK) formulated in Kleptose (15% in 0.1 N NaOH, Roquette, Lestrem, France). This formulation was selected for effectiveness in suppressing SIV replication in the RM model.^25^

Of note, 1 RM (RM23-0168) was found to be not productively infected with SIV following 1 inoculation with 50 TCID50 SIVmac239. Therefore, 4 wk after this initial inoculation, RM23-0168 was inoculated with a second, higher dose of SIVmac239 (100 TCID50), which resulted in productive SIV infection, followed by ART initiation at week 14 p.i. for this animal, which continued through week 20 p.i. In all analyses, data for RM23-0168 is grouped with all other RMs, with week 0 p.i. delineated as the time of the productive second inoculation.

### Sample collection and processing

EDTA and serum gel vacutainer tubes (Sarstedt, Newton, North Carolina, USA) were used to collect peripheral blood. Complete blood counts (CBCs) were performed using EDTA blood on a Sysmex XN-1000v (Sysmex, Kobe, Hyogo, Japan) and blood chemistry was performed using serum on a Beckman AU480 (Beckman, Brea, California, USA). For experimental procedures, EDTA blood was centrifuged for 10 min, 465 × g at room temperature (RT) to isolate plasma, which was stored at −80°C. After plasma removal, whole blood was reconstituted with phosphate buffered saline (PBS; Invitrogen, Carlsbad, California, USA) and 250 µl aliquots of PBS-whole blood were set aside for flow cytometric staining. Peripheral blood mononuclear cells (PBMCs) were isolated from remaining blood via density-gradient centrifugation using Ficoll-Paque Plus (Sigma-Aldrich, St Louis, Missouri, USA). PBMCs were cryopreserved in freezing media (10% dimethyl sulfoxide (DMSO) [Sigma-Aldrich] in heat inactivated fetal bovine serum [Thermo Fisher Scientific, Waltham, Massachusetts, USA]) and stored in liquid nitrogen.

### Flow cytometry

Multicolor flow cytometric analysis was performed on whole blood according to standard procedures and using RM cross-reactive monoclonal antibodies. Samples were first stained at RT with LIVE/DEAD^TM^ Fixable Blue Dead Cell Stain (Thermo Fisher Scientific) for 5 min, then directly treated with BD Pharmingen^TM^ Human Fc Block^TM^ (BD Biosciences, Franklin Lakes, New Jersey, USA) for 10 min at RT. Then, samples were chemokine stained (CCR5) and incubated at 37°C for 10 min. Next, extracellular staining was performed using titrated fluorochrome conjugated antibody concentrations (Table S1, Fig. S1), followed by red blood cell lysis using fluorescence-activated cell sorting (FACS) lysing solution (BD Biosciences). Cells were fixed and permeabilized (CytoFix/CytoPerm^TM^ Fixation/Permeabilization Kit, BD Biosciences), then intracellularly stained (Table S1, Fig. S1). Samples were fixed overnight with 1% paraformaldehyde and held at 4°C until acquisition.

Phagocytosis was evaluated using *E. coli* bioparticle dye conjugates that fluoresce within the acidic environments of endosomes and lysosomes (pHrodo Red *E. coli* Bioparticles Phagocytosis Kit for Flow Cytometry; Thermo Fisher Scientific). Frozen pooled plasma from uninfected RMs was thawed, and pHrodo bioparticles were incubated with this pooled plasma (1:3 plasma: pHrodo volume ratio) for 30 min to allow for bioparticle opsonization. Opsonized pHrodo bioparticles were then added to 250 µl PBS-whole blood aliquots and incubated at 37°C for 2 hr. Samples were stained as described above (Table S1, Fig. S2). Samples were fixed overnight with 1% paraformaldehyde and held at 4°C until acquisition.

Acquisition was performed on a BDSymphony (BD FACSDiva Software v9.3.1) or BD LSRFortessa (BD FACSDiva Software v9.0). Analysis was performed using FlowJo (version 10; BD Biosciences). In all analyzed data, individual cell subsets with less than 100 events in the parent gate were excluded in downstream analyses due to an inability to confidently place gates.

CD4^+^ and CD8^+^ T lymphocyte absolute counts were assessed by staining a 50 µl aliquot of whole blood for 20 min at RT with previously optimized fluorochrome conjugated antibodies (Table S1), followed by lysis with 1× lysis buffer, made from 10× stock (BD Biosciences), for 30 min at RT. Samples were acquired on a MACSQuant 16 (Miltenyi Biotech, Bergisch Gladbach, Germany).

### Detection of plasma markers of neutrophil function

Commercially available enzyme-linked immunosorbent assay (ELISA) kits were used to quantify plasma concentrations of biomarkers of neutrophil extracellular trap formation, including neutrophil elastase (Lifespan Biosciences, Lynnwood, Washington, USA) and citrullinated histone 3 (Cayman Chemicals, Ann Arbor, Michigan, USA), and biomarkers of neutrophil degranulation, including myeloperoxidase (Abcam, Cambridge, UK) and proteinase-3 (MyBioSource, San Diego, California, USA) per the manufacturers’ recommended protocols.

### Data and statistical analysis

Statistical analyses were performed using GraphPad Prism statistical software (Version 10; GraphPad Software, San Diego, California, USA). Statistical significance between all timepoints was calculated using a mixed-effects analysis with the Geisser-Greenhouse correction and a Tukey’s multiple comparisons test, with individual variances computed for each comparison. All reported *P* values were multiplicity adjusted and values of <0.05 were considered significant. Correlations between parameters were calculated using Spearman’s rank correlation test, with Spearman’s rank correlation coefficient (ρ) and *P* values computed for each correlation.

To examine neutrophil phagocytosis, the absolute number of neutrophils/µl blood was used to calculate the number of neutrophils positive for pHrodo bioparticles (phagocytic score, or number of neutrophils capable of phagocytosis).^35^ The phagocytosis score was multiplied by the mean fluorescence intensity (MFI) of pHrodo positive neutrophils to calculate the phagocytic index, which represents phagocytic proficiency.^36^

Data collected from CBCs with differentials were utilized to calculate hematological parameters as follows: systemic immune inflammation (SII) index = neutrophils/lymphocytes × platelets; platelet-to-neutrophil ratio (PNR) = platelets/neutrophils; neutrophil-to-monocyte ratio (NMR) = neutrophils/monocytes; and neutrophil-to-lymphocyte ratio (NLR) = neutrophils/lymphocytes.^37–40^ CBC data also yielded hematocrit values at each timepoint, which were used to determine anemia, defined as below 34.8% hematocrit.^41^

## Results

### Experimental design

**Figure 1.**
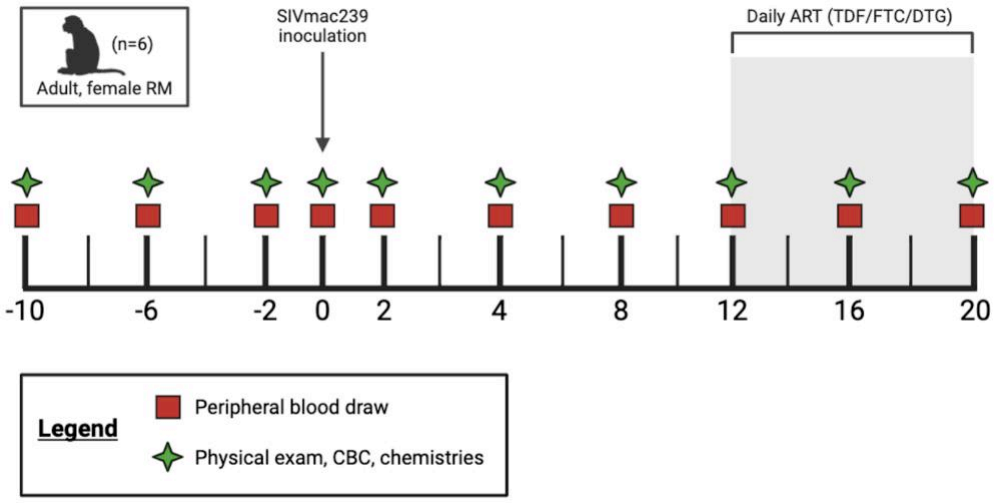
Experimental timeline. Experimental timeline depicting sample collection from adult female rhesus macaques (RMs, n = 6). Baseline samples were taken at −10, −6, −2, and 0 wk prior to SIV inoculation. Samples were also collected at weeks 2, 4, 8, 12, 16, and 20 post-infection (p.i.). Red squares indicate peripheral blood draws. Green stars indicate physical exam, complete blood count (CBC), and serum chemistries. Daily antiretroviral therapy (ART) was initiated at week 12 p.i. and consisted of tenofovir disoproxil fumarate (TDF; 5.1 mg/kg), emtricitabine (FTC; 30 mg/kg) and dolutegravir (DTG; 2.5 mg/kg).

Adult, female Indian-origin RMs (n = 6) were used to characterize links between peripheral neutrophil frequency and function with systemic markers of inflammation and immune activation during acute SIV infection and in the context of ART. Demographic information of RMs enrolled in this study are listed in Table 1, which includes anonymized animal ID, sex, age, weight prior to study enrollment, geographic origin, and MHC haplotypes. Blood draws and physical exams were conducted at 3 baseline timepoints, as well as throughout acute SIV infection and ART (Fig. 1). Measurements at weeks −10, −6, −2, and 0 were averaged and reported as “baseline” values. At week 0, RMs were intravenously inoculated with SIVmac239 (50 TCID50). Daily ART (TDF/FTC/DTG) was initiated at week 12 post-inoculation (p.i.) and continued through week 20 p.i.

### SIV dynamics and shifts in hematological parameters during acute and ART-treated SIV infection

**Figure 2.**
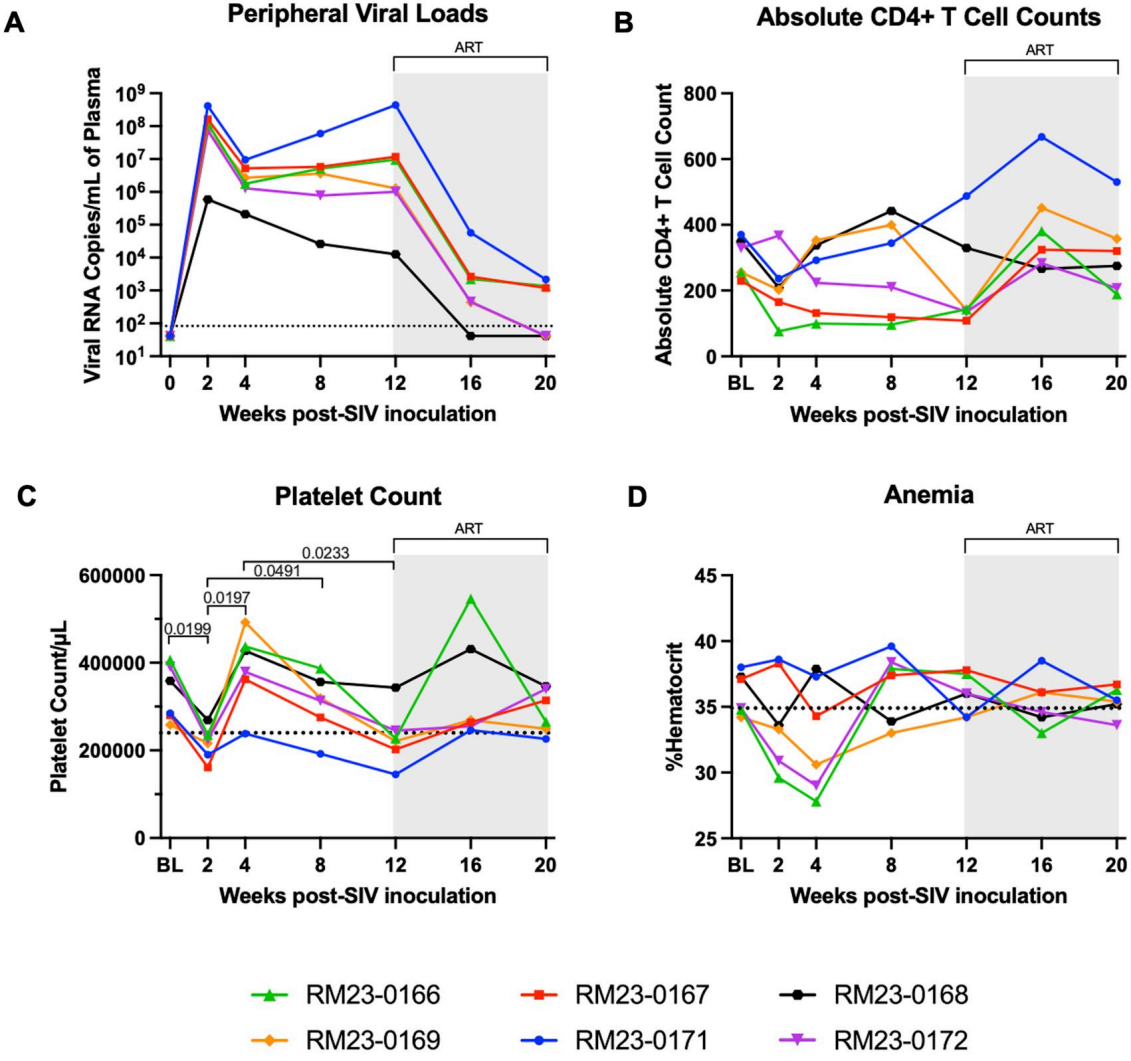
Clinical signs of HIV/SIV infection established during acute SIV-infection of adult female rhesus macaques. Peripheral SIVmac239 viral load (VL), blood CD4^+^ T cell count, platelet count (PLT), and hematocrit (HCT) levels were assessed in adult female rhesus macaques (RMs; n = 6). (A) Plasma viral load (RNA copies/mL plasma) was assessed via RT-qPCR. Dotted line at 10^2^ represents undetectable levels of viral RNA. Values below the limit of detection (83 copies/ml) are displayed as half the limit of detection (41.5 copies/ml). (B) Absolute number of CD4^+^ T cells per µl of blood was assessed via flow cytometry using the antibodies in Table S1. (C) Platelet count (PLT/µl) was assessed by complete blood count. Dotted line at 240,000 PLT/µl represents threshold for thrombocytopenia in rhesus macaques. (D) Hematocrit (HCT) was assessed by complete blood count. Dotted line at 34.9% represents threshold for anemia previously reported. In all panels, each RM is represented by different color and symbol. Intravenous inoculation with SIVmac239 occurred at week 0. Baseline measurements at weeks −10, −6, −2, and 0 were averaged and are represented as “BL.” Daily antiretroviral therapy (ART) was initiated at week 12 p.i., indicated by the light gray bar. Statistical significance between all timepoints was computed using a mixed-effects analysis with the Geisser-Greenhouse correction and Tukey’s multiple-comparison test, with individual variances calculated for each comparison. Significant multiplicity-adjusted *P* values are shown above horizontal black brackets.

We first characterized peripheral viral dynamics during SIVmac239 infection by quantifying plasma viral loads (VL) via quantitative reverse transcription PCR, as previously described^34^ and quantifying the absolute number of peripheral CD4+ T cells via flow cytometry (Fig. 2; Table S1). All RMs reached peak viremia within 2 wk p.i., with a median viral load of 98,350,000 copies/mL plasma (range = 588,000 to 414,000,000 copies/ml; Fig. 2A). All RMs maintained high plasma viremia throughout the acute stage of infection (weeks 2 through 12 p.i.; Fig. 2A). ART administration beginning at week 12 p.i. led to a reduction in plasma viremia by week 16 p.i., with n = 1 animal becoming undetectable at week 16 p.i. and n = 2 animals undetectable at week 20 p.i. (Fig. 2A). Following SIV inoculation, n = 5 RMs experienced a decrease in the absolute number of peripheral CD4^+^ T cells at week 2 p.i. compared to baseline, however this decline did not reach statistical significance (Fig. 2B). Throughout the remainder of acute SIV infection, minimal disruptions in peripheral absolute CD4^+^ T cell counts were observed, with similar numbers of CD4^+^ T cells per µl of blood detected over time (Fig. 2B). Finally, following ART-initiation, n = 5 RMs exhibited elevations in peripheral absolute CD4^+^ T cell count as compared to acute SIV infection, although not significant (Fig. 2B).

Additional clinical hallmarks of HIV/SIV infection include thrombocytopenia and anemia.^41–43^ Therefore, we used complete blood count (CBC) data to assess platelet (PLT) counts and hematocrit (HCT) levels during both acute SIV infection and ART-treated SIV infection. Median PLT counts were significantly lower at week 2 p.i. as compared to baseline (*P* = 0.0199; Fig. 2C). At week 2 p.i., n = 4 RMs had PLT counts below the threshold for thrombocytopenia in RMs (240,000 PLT/µl; Fig. 2C). Median PLT counts increased concurrently with establishment of viral set point and were significantly higher at week 4 and 8 p.i. as compared to week 2 p.i. (*P* = 0.0197 and *P* = 0.0491, respectively; Fig 2C). Median PLT counts declined over time and were significantly lower at week 12 p.i. compared to week 4 p.i. (*P* = 0.0233; Fig. 2C). Anemia, defined as the percent HCT below the established anemia threshold of 34.8% for both timepoints,^41^ was observed in n = 4 RMs at peak viremia (week 2 p.i.) and n = 4 RMs at week 4 p.i. (Fig. 2D). By week 8 p.i., percent HCT increased above the anemia threshold in all but 2 RMs. In the context of ART, most RMs were not anemic, but values fluctuated around the clinical anemia threshold. Taken together, these data demonstrate that classic clinical signs of pathogenic HIV/SIV infection, including high SIV VL, lowered absolute CD4^+^ T cell counts, and disruptions in PLT counts and HCT levels, were observed in this cohort of adult female SIVmac239-infected RMs and were restored to baseline levels following ART initiation.

### Reductions in peripheral CCR5^+^ CD4^+^ T cells and expansions of Ki-67^+^ CD4^+^ T cells in the periphery during acute SIV infection are restored to baseline levels following ART

**Figure 3.**
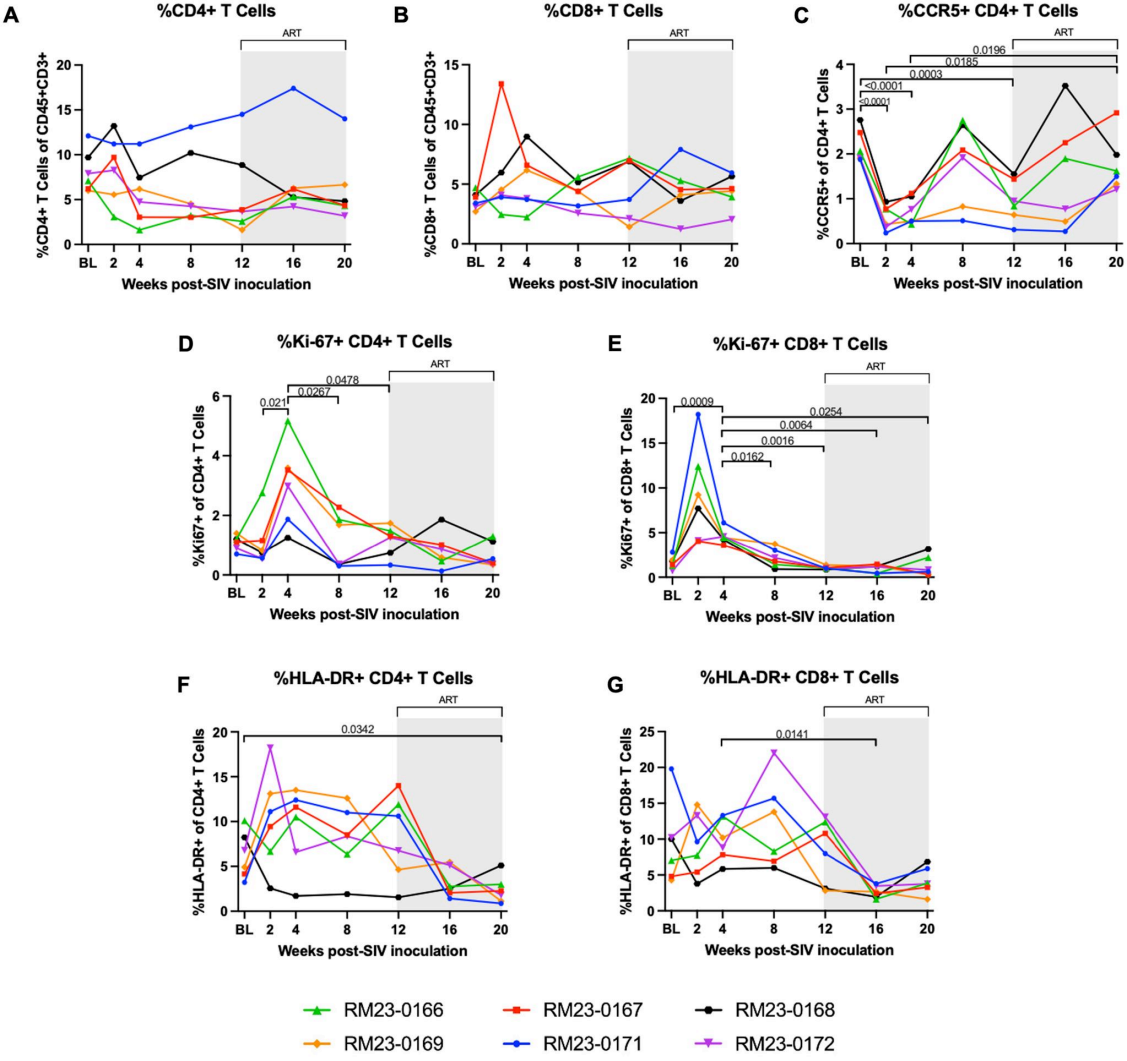
Shifts in peripheral CD4^+^ and CD8^+^ T cell kinetics during acute SIV infection and during ART. Frequencies of CD4^+^ T cells (A) and CD8^+^ T cells (B) of CD45^+^ cells in peripheral blood was assessed by flow cytometry. Frequencies of CCR5^+^ CD4^+^ T cells (C), Ki-67^+^ CD4^+^ T cells (D), Ki-67^+^ CD8^+^ T cells (E), HLA-DR^+^ CD4^+^ T cells (F), and HLA-DR^+^ CD8^+^ T cells (G) were also quantified by flow cytometry. In all panels, each RM is represented by different color and symbol. Baseline measurements at weeks −10, −6, −2, and 0 were averaged and are represented as “BL.” Daily antiretroviral therapy (ART) was initiated at week 12 p.i., indicated by the light gray bar. Statistical significance between all timepoints was computed using a mixed-effects analysis with the Geisser-Greenhouse correction and Tukey’s multiple-comparison test, with individual variances calculated for each comparison. Significant multiplicity-adjusted *P* values are shown above horizontal black brackets.

We next characterized total CD4^+^ and CD8^+^ T cell frequencies, defined as viable CD45^+^ CD3^+^ CD4^+/−^ and CD8^+/−^ cells, as well as the frequency of T cells expressing CCR5, an HIV/SIV co-receptor,^44^ Ki-67, a marker of cell proliferation and activation,^45^ and HLA-DR, which has been used as a marker of immune activation^46^ throughout acute SIV infection and ART via flow cytometry. No significant differences in peripheral CD4^+^ T cell frequencies were observed over time during SIV infection and ART (Fig. 3A). However, we did observe significant depletion of CCR5^+^ CD4^+^ T cells at weeks 2, 4, and 12 p.i. as compared to baseline (*P* < 0.0001, *P* < 0.0001, and *P* = 0.0003, respectively; Fig. 3C). At week 20 p.i., after 2 mo of daily ART, CCR5^+^ CD4^+^ T cells were significantly increased as compared to weeks 2 and 4 p.i. (*P* = 0.0185 and *P* = 0.0196, respectively; Fig. 3C). One possible explanation for why the significant depletion of CCR5^+^ CD4^+^ T cells did not impact overall CD4^+^ T cell frequencies in the periphery during acute SIV infection could be that proliferation of CD4^+^ T cells replenished the CD4^+^ T cell pool, thereby maintaining overall CD4^+^ T cell frequencies. To examine this possibility, we next assessed the frequency of proliferating CD4^+^ T cells by characterizing the frequency of Ki-67 expressing CD4^+^ T cells. Indeed, we observed significantly elevated frequencies of Ki-67^+^ CD4 T cells at week 4 p.i. compared to weeks 2, 8, and 12 p.i. (*P* = 0.0211, *P* = 0.0267 and *P* = 0.0478, respectively; Fig. 3D), all timepoints prior to ART initiation. Finally, we assessed the frequency of activated CD4^+^ T cells by quantifying the frequency of HLA-DR expressing CD4^+^ T cells. At peak viremia (week 2 p.i.), n = 4 animals demonstrated an increase in circulating HLA-DR^+^ CD4^+^ T cells which were sustained through the time of ART initiation (week 12 p.i.; Fig. 3F). After 2 mo of daily ART, we observed a significant depletion of peripheral HLA-DR^+^ CD4^+^ T cells at week 20 p.i. as compared to baseline (*P* = 0.0342; Fig. 3F). Taken together, these data demonstrate that depletion of CCR5^+^ CD4^+^ T cells was restored to baseline levels and elevated frequencies of activated HLA-DR^+^ CD4^+^ T cells were decreased compared to baseline following ART initiation, and that proliferation of peripheral CD4^+^ T cells during acute infection may account for unchanged total CD4^+^ T cell frequencies over time.

### CD8^+^ T cell proliferation and activation are significantly reduced following ART initiation in acute SIV infection

The kinetics of CD8^+^ T cells were also assessed using flow cytometry. Similar to CD4^+^ T cells, we observed no significant changes in peripheral CD8^+^ T cell frequencies throughout infection or in the context of ART (Fig. 3B). At peak viremia (week 2 p.i.), n = 6 animals demonstrated an increase in circulating Ki-67^+^ CD8^+^ T cells compared to baseline, and there was a significant elevation of Ki-67^+^ CD8^+^ T cells at week 4 p.i. compared to baseline (*P* = 0.0009; Fig. 3E). This transient increase in Ki-67^+^ CD8^+^ T cells was followed by significant reductions in these cells at weeks 8, 12, 16, and 20 p.i. compared to week 4 p.i. (*P* = 0.0162, *P* = 0.0016, *P* = 0.0064 and *P* = 0.0254, respectively; Fig. 3E). Thus, after peak viremia, the transient increase in frequencies of peripheral Ki-67^+^ CD8^+^ T cells normalizes back to baseline levels and is sustained to these levels after ART initiation. We also assessed the frequency of activated HLA-DR expressing CD8^+^ T cells via flow cytometry. No significant differences in the frequency of HLA-DR^+^ CD8^+^ T cells were observed throughout acute SIV infection, however there was a significant depletion of peripheral HLA-DR^+^ CD8^+^ T cells after 1 mo of ART compared to week 4 p.i. (*P* = 0.0141; Fig. 3G). In sum, consistent with previous studies,^45^^,^^47–49^ our data indicate that CD8^+^ T cell proliferation is elevated during acute SIV infection, and that both CD8^+^ T cell proliferation and activation are restored to baseline levels following ART initiation.

### Shifts in hematological markers of systemic inflammation occur during acute SIV infection and are restored to baseline levels after ART

**Figure 4.**
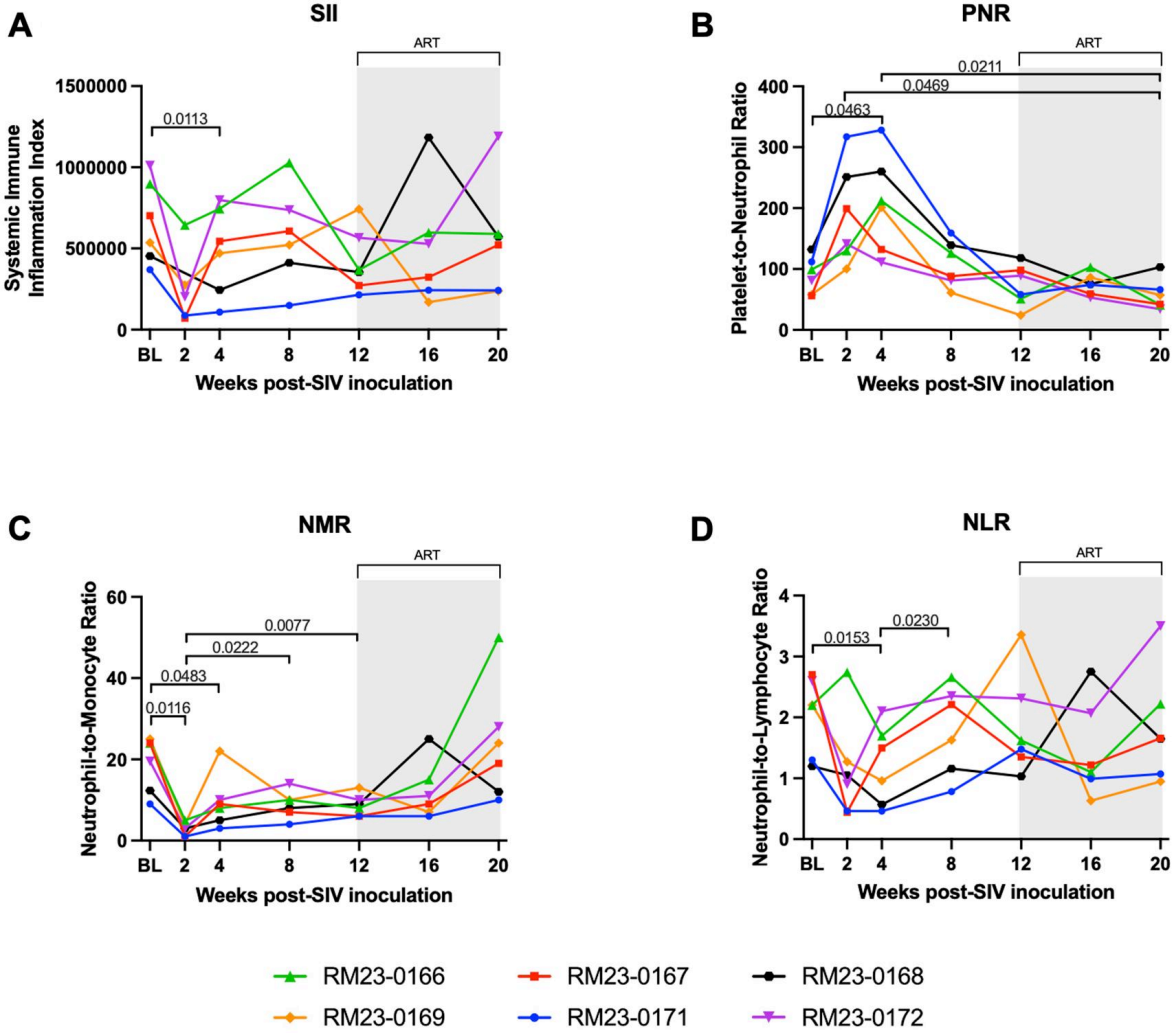
Hematological markers of inflammation during acute SIV infection and in the context of persistent, daily ART in female rhesus macaques. (A) Systemic immune-inflammation index (SII) was obtained from complete blood chemistry (CBC) values. SII was calculated with the following formula: SII = (platelet count * neutrophil count)/lymphocyte count. (B) Platelet-to-neutrophil ratio (PNR) was obtained from CBC values. PNR was calculated with the following formula: PNR = platelet count/neutrophil count. (C) Neutrophil-to-monocyte ratio (NMR) was obtained from CBC values. NMR was calculated with the following formula: NMR = neutrophil count/monocyte count. (D) Neutrophil-to-lymphocyte ratio (NLR) was obtained from CBC values. NLR was calculated with the following formula: NLR = neutrophil count/lymphocyte count. In all panels, each RM is represented by different color and symbol. Baseline measurements at weeks −10, −6, −2, and 0 were averaged and are represented as “BL.” Antiretroviral therapy (ART) was initiated at 12 w.p.i., indicated by the light gray bar. Statistical significance between all timepoints was computed using a mixed-effects analysis with the Geisser-Greenhouse correction and Tukey’s multiple-comparison test, with individual variances calculated for each comparison. Significant multiplicity-adjusted *P* values are shown above horizontal black brackets.

To further characterize the impact of acute SIV infection and ART on indicators of systemic inflammation, we assessed several hematological parameters that have been shown to correlate with systemic inflammation and the innate immune response. The systemic immune-inflammation (SII) index is a marker of inflammatory and immune status based on neutrophil, lymphocyte, and platelet counts in peripheral blood.^37^ SII is reflective of systemic inflammation and the balance between the innate and adaptive immune response.^50^ In this cohort of RMs, we observed a significant decrease in SII value at week 4 p.i. compared to baseline (*P* = 0.0113) followed by a return to baseline levels during viral set point, which were maintained throughout ART (Fig. 4A).

The platelet-to-neutrophil ratio (PNR) is another hematological parameter of systemic inflammation.^38^ We observed a significant increase in the PNR at week 4 p.i. compared to baseline (*P* = 0.0463), followed by a decline in the PNR values by weeks 8 and 12 p.i. (Fig. 4B). At weeks 8 and 12 p.i., after acute infection and prior to ART initiation, the PNR values normalized to a level similar to BL levels (Fig. 4B). After two months of daily ART, there was a significant reduction in the median PNR at week 20 p.i. compared to weeks 2 and 4 p.i. (*P* = 0.0464 and *P* = 0.0211, respectively; Fig. 4B).

The neutrophil-to-monocyte ratio (NMR) is an additional hematological marker of systemic inflammation which considers the proportion of neutrophils and monocytes in peripheral blood. Prior work has suggested that persistent, low levels of inflammation are associated with higher NMR values, due to increased neutrophils and decreased monocytes.^39^ Compared to baseline values, there was a significant decrease in the NMR at weeks 2 and 4 p.i. (*P* = 0.0016 and *P* = 0.0483, respectively; Fig. 4C). The NMR was increased at weeks 8 and 12 p.i. compared to week 2 p.i. (*P* = 0.0222 and *P* = 0.0077, respectively) and remained relatively stable throughout ART (Fig. 4C).

Finally, the neutrophil-to-lymphocyte ratio (NLR) is an indicator of the balance between acute and chronic inflammation and the adaptive immune response.^40^ Significant changes in NLR over time could be indicative of disruption in immune system homeostasis.^40^ In clinical settings, prognostic NLR values are typically characterized by an increase in neutrophils and a decline in lymphocytes.^51^ We observed a significant decrease in the NLR at week 4 p.i. compared to baseline (*P* = 0.0153), and a significant increase in the NLR at week 8 p.i. as compared to week 4 p.i. (*P* = 0.0230; Fig. 4D). However, no significant changes in the NLR were observed during ART. Taken together, these data on hematological parameters of inflammation, including SII index, PNR, NMR, and NLR demonstrate systemic inflammation reflective of disruption of innate and adaptive immune responses begin to resolve after acute SIV infection and are maintained with daily ART.

### Peripheral neutrophil frequencies are depleted in early SIV infection with a return to baseline levels during ART

**Figure 5.**
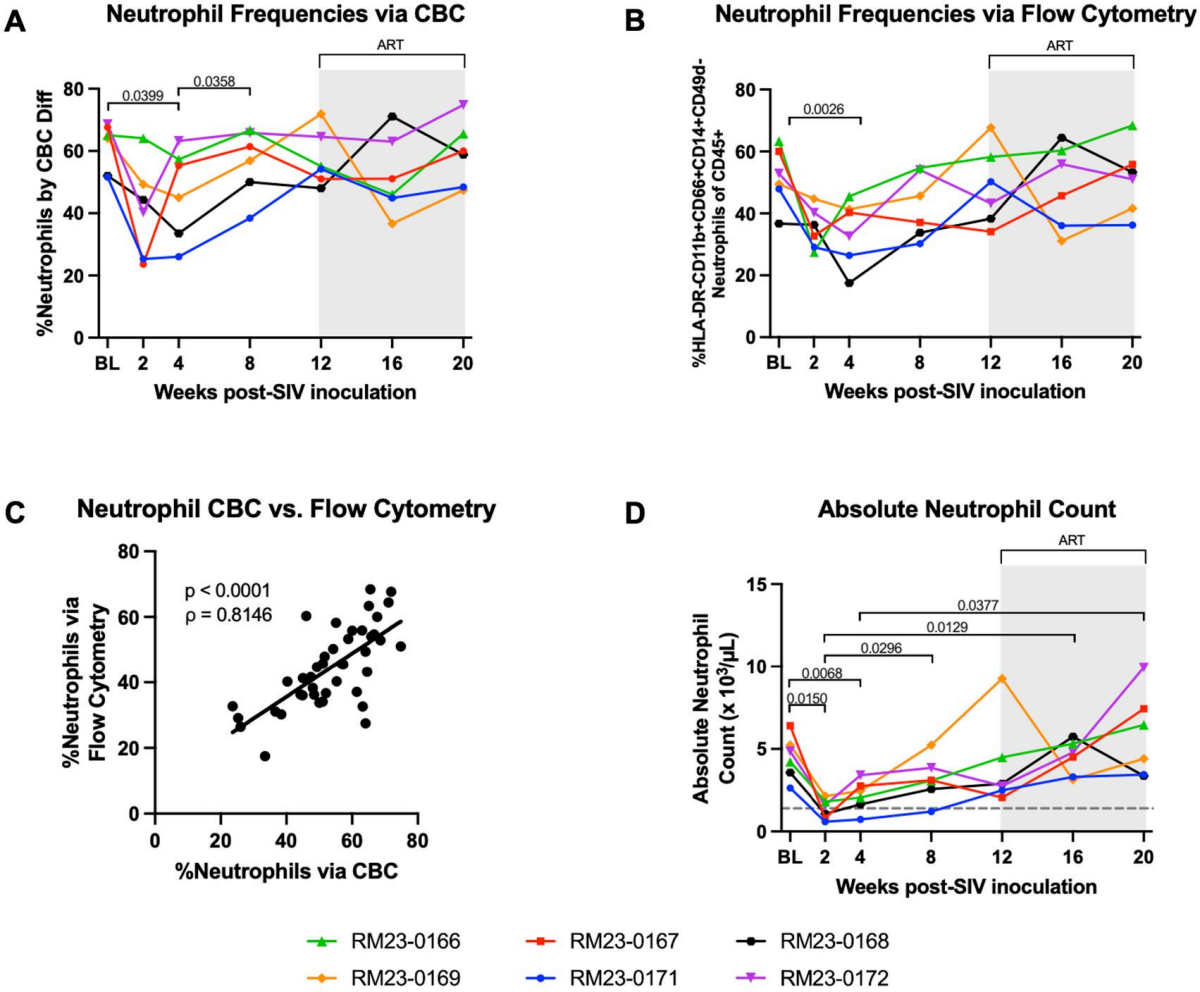
Depletion of peripheral neutrophils during acute SIV infection is restored to baseline levels after ART initiation. (A) Neutrophil frequencies quantified by complete blood count (CBC) with differential measures. (B) Neutrophil frequencies quantified by flow cytometry. (C) Comparison of % neutrophils in rhesus macaque whole blood obtained from flow cytometry vs. CBC with differential measures. Neutrophil frequencies as a % of total CD45^+^ cells obtained via flow cytometry is represented on the *y* axis. These data were then correlated with % neutrophils quantified by CBC with differential measures, represented on the *x* axis. Significance was assessed using the Spearman’s rank correlation test. (D) Absolute peripheral neutrophil count (× 10^3^/µl) quantified by complete blood count (CBC) with differential measures. Gray dashed line represents threshold for neutropenia (1.4 × 10^3^/µl). In all panels, each RM is represented by different color and symbol. Intravenous inoculation with SIVmac239 occurred at week 0. Baseline measurements at weeks −10, −6, −2, and 0 were averaged and are represented as “BL.” Antiretroviral therapy (ART) was initiated at 12 w.p.i., indicated by the light gray bar. Statistical significance between all timepoints was computed using a mixed-effects analysis with the Geisser-Greenhouse correction and Tukey’s multiple-comparison test, with individual variances calculated for each comparison. Significant multiplicity-adjusted *P* values are shown above horizontal black brackets.

Given that our assessment of hematological parameters of inflammation indicated that disruptions of neutrophils relative to platelets, monocytes, and lymphocytes occurred in the context of both SIV infection and ART, we next sought to characterize neutrophil responses to unravel the potential role of neutrophils in SIV-associated inflammation. First, we assessed the percentage of neutrophils in peripheral blood quantified by CBC differential (Fig. 5A). We observed a significant reduction in neutrophil percentages at week 4 p.i. (*P* = 0.0399) compared to baseline and a significant increase in neutrophil percentages at week 8 p.i. compared to week 4 p.i. (*P* = 0.0358; Fig. 5A). To confirm these findings, we also characterized peripheral neutrophil frequencies utilizing flow cytometry. Neutrophils were identified as viable CD45^+^ HLA-DR^−^ CD11b^+^ CD66abce^+^ CD14^+^ CD49d^−^ cells, as previously described (Fig. S1).^15^^,^^52–56^ Consistent with data from the CBC differential, we observed a significant decline in peripheral neutrophils at week 4 p.i. compared to baseline (*P* = 0.0026; Fig 5B). Neutrophil frequencies returned to baseline levels and were unchanged throughout ART as compared to baseline when assessed by both CBC differential and flow cytometry. As reported previously,^57^ neutrophil frequencies observed via flow cytometry significantly correlated with values obtained by CBC differential, thereby validating our flow cytometric approach to identify neutrophils in whole blood (Spearman’s rank correlation rho = 0.8146; *P* < 0.0001; Fig. 5C). Finally, the absolute counts of neutrophils (×10^3^/µl) in whole blood were quantified by CBC with differential measures. We observed significant decrease in peripheral neutrophil counts in all RMs at week 2 p.i. compared to baseline (*P* = 0.0150; Fig. 5D), with n = 3 RMs dropping below the threshold for neutropenia (≤1.4 × 10^3^/µl). This depletion of peripheral neutrophil counts was restored by week 8 p.i. (*P* = 0.0296; Fig. 5D). In sum, these data indicate that intravenous SIVmac239 inoculation induces transient neutropenia, reflected by absolute counts and flow cytometric characterization, which begins to restore later in acute infection and continues to rise in the context of ART.

### Markers of neutrophil apoptosis and neutrophil extracellular trap formation are elevated during acute SIV infection and are restored to baseline levels after ART

**Figure 6.**
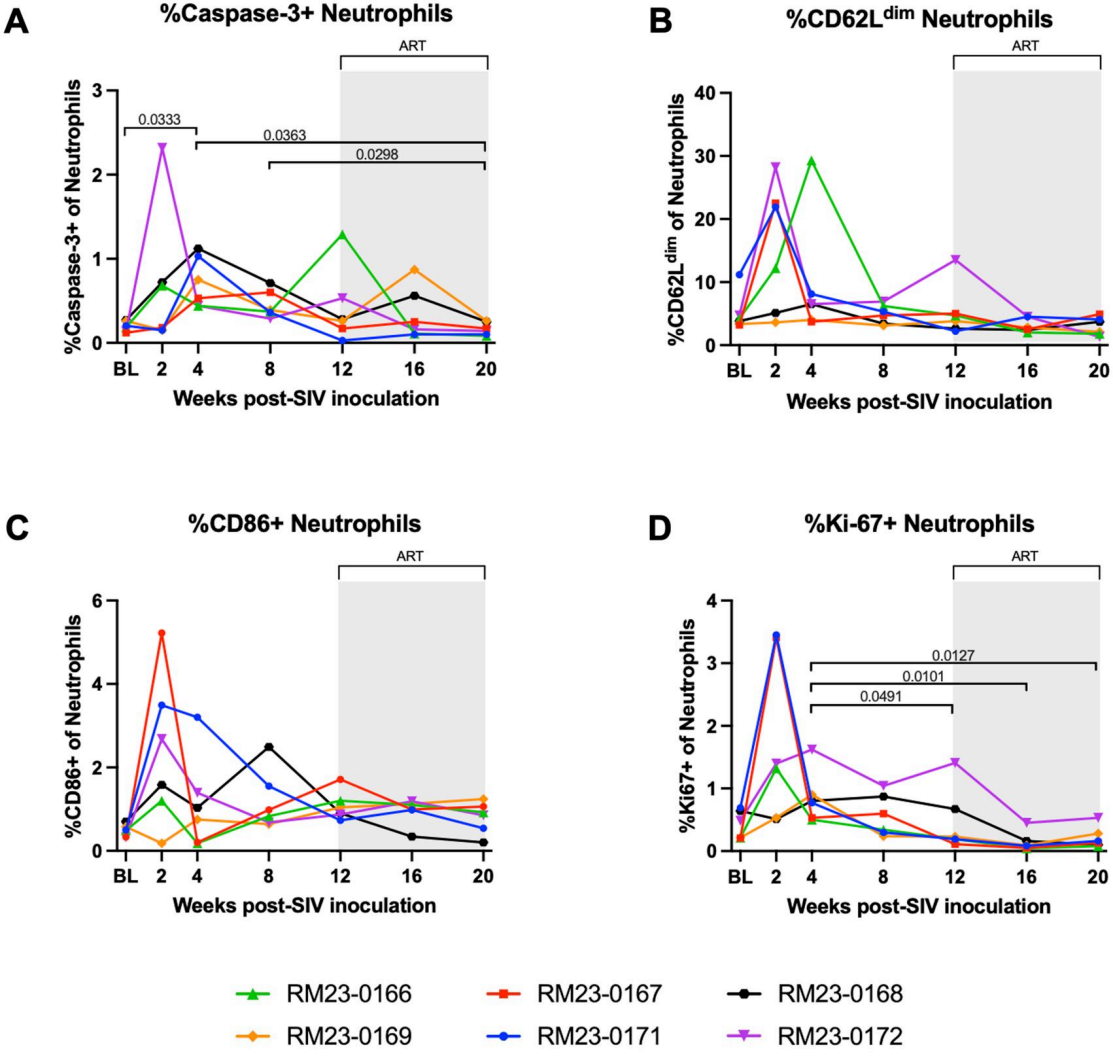
Increased neutrophil apoptosis and indication of neutrophil extracellular trap formation are observed during acute SIV infection, but are restored to baseline levels after ART. Percent of peripheral neutrophils expressing caspase-3 (A), CD62L (B), CD86 (C), and Ki67 (D) quantified by flow cytometry. In all panels, each RM is represented by different color and symbol. Intravenous inoculation with SIVmac239 occurred at week 0. Baseline measurements at weeks −10, −6, −2, and 0 were averaged and are represented as “BL.” Daily antiretroviral therapy (ART) was initiated at week 12 p.i., indicated by the light gray bar. Statistical significance between all timepoints was computed using a mixed-effects analysis with the Geisser-Greenhouse correction and Tukey’s multiple-comparison test, with individual variances calculated for each comparison. Significant multiplicity-adjusted *P* values are shown above horizontal black brackets.

To characterize peripheral neutrophil phenotype throughout SIV infection and ART, we first used flow cytometry to assess intra- and extracellular expression of apoptosis and activation markers (Fig. S1, Table S1). Caspase-3 mediated apoptosis is a common mechanism of neutrophil clearance,^58^ and previous work has demonstrated increased neutrophil apoptosis during acute, pathogenic SIV infection.^59^ Increased neutrophil apoptosis is also associated with dysfunction and progression to AIDS.^59^ Thus, we sought to examine neutrophil apoptosis via expression of caspase-3 (Fig. 6A). There was a significant increase in the frequency of caspase-3^+^ neutrophils at week 4 p.i. as compared to baseline (*P* = 0.0333), after which the frequency of caspase-3^+^ neutrophils returned to baseline levels (Fig. 6A). Interestingly, after two months of daily ART, the frequency of caspase-3^+^ neutrophils was significantly decreased as compared to weeks 4 and 8 p.i. (*P* = 0.0363 and *P* = 0.0298, respectively; Fig. 6A). Taken together, these data demonstrate increased apoptosis of peripheral neutrophils during acute SIV infection followed by greater neutrophil survival during ART.

Another feature of neutrophil status is their maturation and priming, which is associated with surface expression of CD62L.^60^ Interestingly, a sub-population of CD62L^dim^ neutrophils has been shown to be recruited to the bloodstream during acute inflammation,^61^ particularly during acute SIV infection.^62^ Thus, we sought to assess CD62L expression to gain insight to neutrophil maturation and recruitment during acute SIV and following ART. Although we observed a transient increase in peripheral CD62L^dim^ neutrophils in n = 4 animals at week 2 p.i., this increase was not statistically significant, and no differences were observed in the frequency of CD62L^dim^ neutrophils following ART as compared to baseline (Fig. 6B).

Neutrophils are critical to the innate immune response, but they are traditionally viewed as short-lived effector cells.^63^ However, neutrophils may also play an immunoregulatory role by suppressing T cell proliferation or promoting T cell activation through co-stimulatory molecules.^64^^,^^65^ Several studies have demonstrated that neutrophils can express CD86, a key co-stimulatory molecule capable of modulating the regulatory T cell response.^66–69^ Here, we observed a transient increase in CD86^+^ neutrophils in n = 5 RMs during peak viremia (median = 2.68%) compared to baseline (median = 0.49%; Fig. 6C). Following ART, no differences were observed in the frequency of CD86^+^ neutrophils as compared to baseline (Fig. 6C).

Previous work by Amulic et al. used purified human peripheral blood neutrophils to demonstrate that resting neutrophils do not express Ki-67, while neutrophils stimulated in vitro to undergo neutrophil extracellular trap (NET) formation transiently express Ki-67.^70^ Here, flow cytometric staining of neutrophils for Ki-67 expression demonstrated a significantly higher frequency of Ki-67^+^ neutrophils during acute infection at week 4 p.i. as compared to weeks 12, 16, and 20 p.i. (*P* = 0.0491, *P* = 0.0101, and *P* = 0.0127, respectively; Fig. 6D). In sum, these findings demonstrate that acute SIV infection impacts the frequency of peripheral neutrophils expressing markers indicative of apoptosis and NET formation, and that these SIV-induced disruptions are restored to baseline levels following ART.

### Minimal disruptions in peripheral biomarkers of NET formation during acute SIV and early ART

**Figure 7.**
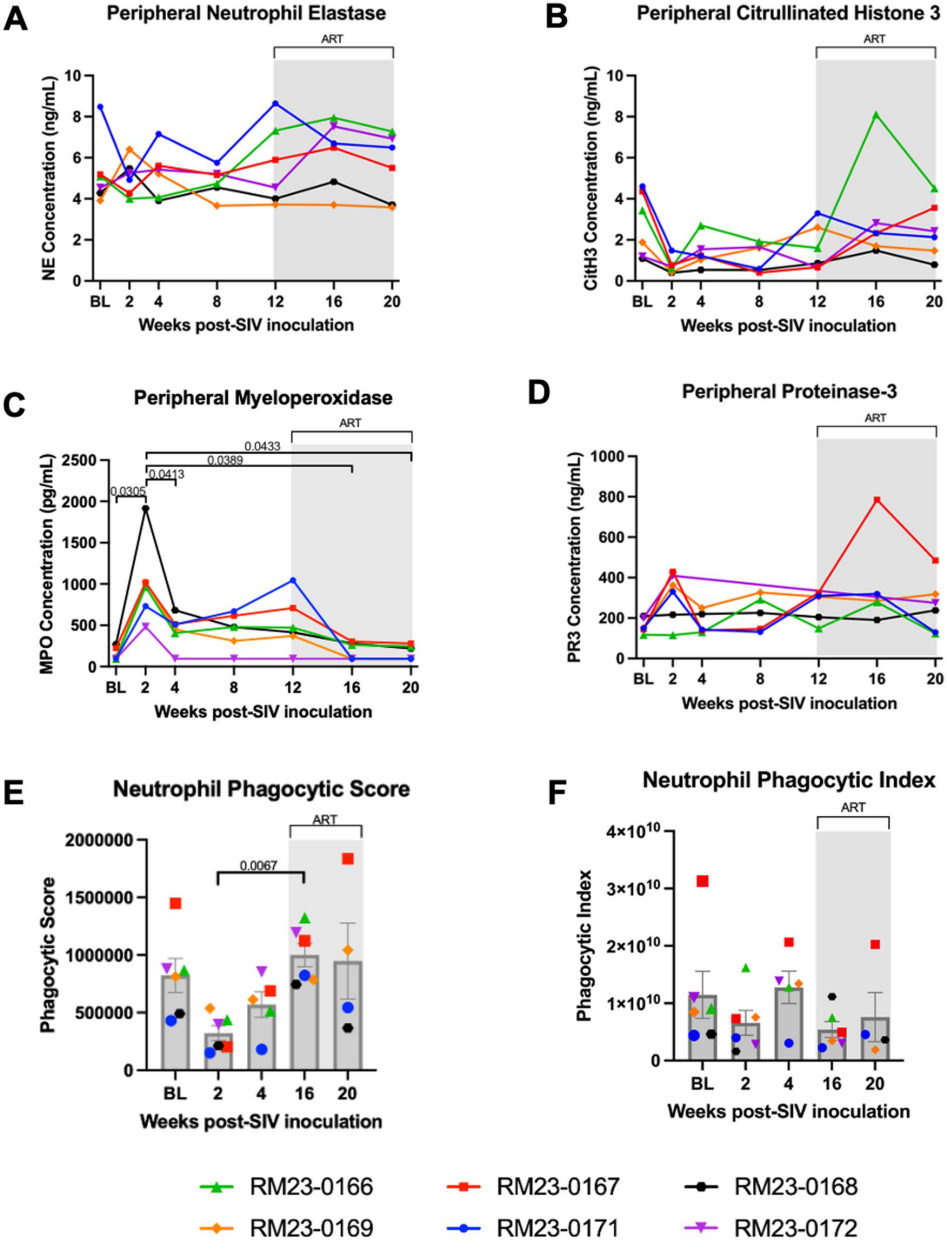
Transient increase in myeloperoxidase and decrease in neutrophil phagocytic ability during acute SIV infection is restored after early ART initiation. Biomarkers of neutrophil extracellular trap formation (A) neutrophil elastase and (B) citrullinated histone 3, and biomarkers of neutrophil degranulation, (C) myeloperoxidase and (D) proteinase-3 in peripheral plasma measured by ELISA. (E) Neutrophil phagocytic score was calculated by the formula: [absolute number of neutrophils/µl of whole blood] * [% of neutrophils positive for uptake of pHrodo bioparticle]. (F) Neutrophil phagocytic index was calculated by the formula: [phagocytic score] * [median fluorescence intensity of pHrodo positive neutrophils]. Intravenous inoculation with SIVmac239 occurred at week 0. Baseline measurements at weeks -10, -6, -2, and 0 were averaged and are represented as “BL.” Antiretroviral therapy (ART) was initiated at week 12 p.i., indicated by the light gray bar. Statistical significance between all timepoints was computed using a mixed-effects analysis with the Geisser-Greenhouse correction and Tukey’s multiple-comparison test, with individual variances calculated for each comparison. Significant multiplicity-adjusted *P* values are shown above horizontal black brackets.

Prior work has demonstrated that chronic HIV infection induces neutrophil extracellular trap (NET) formation in gut, lung, liver, and heart vasculature, and that ART may reduce these levels of NETosis.^30^^,^^71^ Given these prior findings and our observation of a transient increase in Ki-67^+^ neutrophils, which identifies neutrophils that may be poised to undergo NET formation, we next quantified peripheral biomarkers of NET formation by ELISA. Specifically, we evaluated plasma concentrations of neutrophil elastase (NE) and citrullinated histone 3 (CitH3), which are both well-defined surrogate markers of peripheral NET formation.^72–75^ Our data demonstrated no significant changes in plasma levels of NE or CitH3 throughout SIV infection or during ART (Fig. 7A and B, respectively).

### Increase in peripheral neutrophil degranulation observed during peak viremia is significantly reduced after two months of ART

Previous studies have demonstrated that increased neutrophil activation and degranulation are correlated with disease severity in PWH.^76^^,^^77^ While the effect of ART on neutrophil oxidative burst has been previously assessed,^18^ the effect of ART on circulating neutrophil degranulation markers has not been fully elucidated. We thus sought to examine neutrophil degranulation during acute SIV infection and in the context of ART by measuring plasma concentrations of myeloperoxidase (MPO) and proteinase-3 (PR3) via ELISA. We observed a significant increase in peripheral MPO concentrations during peak viremia as compared to baseline and week 4 p.i. (*P* = 0.0305 and *P* = 0.0413, respectively; Fig. 7C). At weeks 16 and 20 p.i., there was a significant decrease in peripheral MPO concentrations compared to week 2 p.i. (*P* = 0.0389 and *P* = 0.0433, respectively; Fig. 7C). No significant changes in PR3 plasma concentrations were noted throughout the experimental timeline (Fig. 7D). These data demonstrate a transient increase in peripheral neutrophil degranulation of MPO during peak viremia, which is restored to baseline levels following viral setpoint and 1 to 2 mo on ART.

### Peripheral neutrophil phagocytic capability declines during acute SIV infection and is restored with ART

Previous work has demonstrated that HIV infection impairs the phagocytic abilities of immune cells, including neutrophils.^78^^,^^79^ Thus, we next investigated whether neutrophil phagocytic abilities are impaired during acute SIV infection, and the impact of ART on restoration of phagocytic function. To assess phagocytic function of neutrophils, we measured phagocytic score (capability) and phagocytic index (proficiency) in peripheral neutrophils via flow cytometry (Table S3). All n = 6 RMs had lower values of neutrophil phagocytic score at peak viremia compared to baseline values, though this difference was not statistically significant (Fig. 7E). Interestingly, phagocytic score was significantly increased at week 16 p.i. as compared to week 2 p.i. (*P* = 0.0067; Fig. 7E). No significant differences in phagocytic index were observed throughout the study (Fig. 7F).

**Figure 8.**
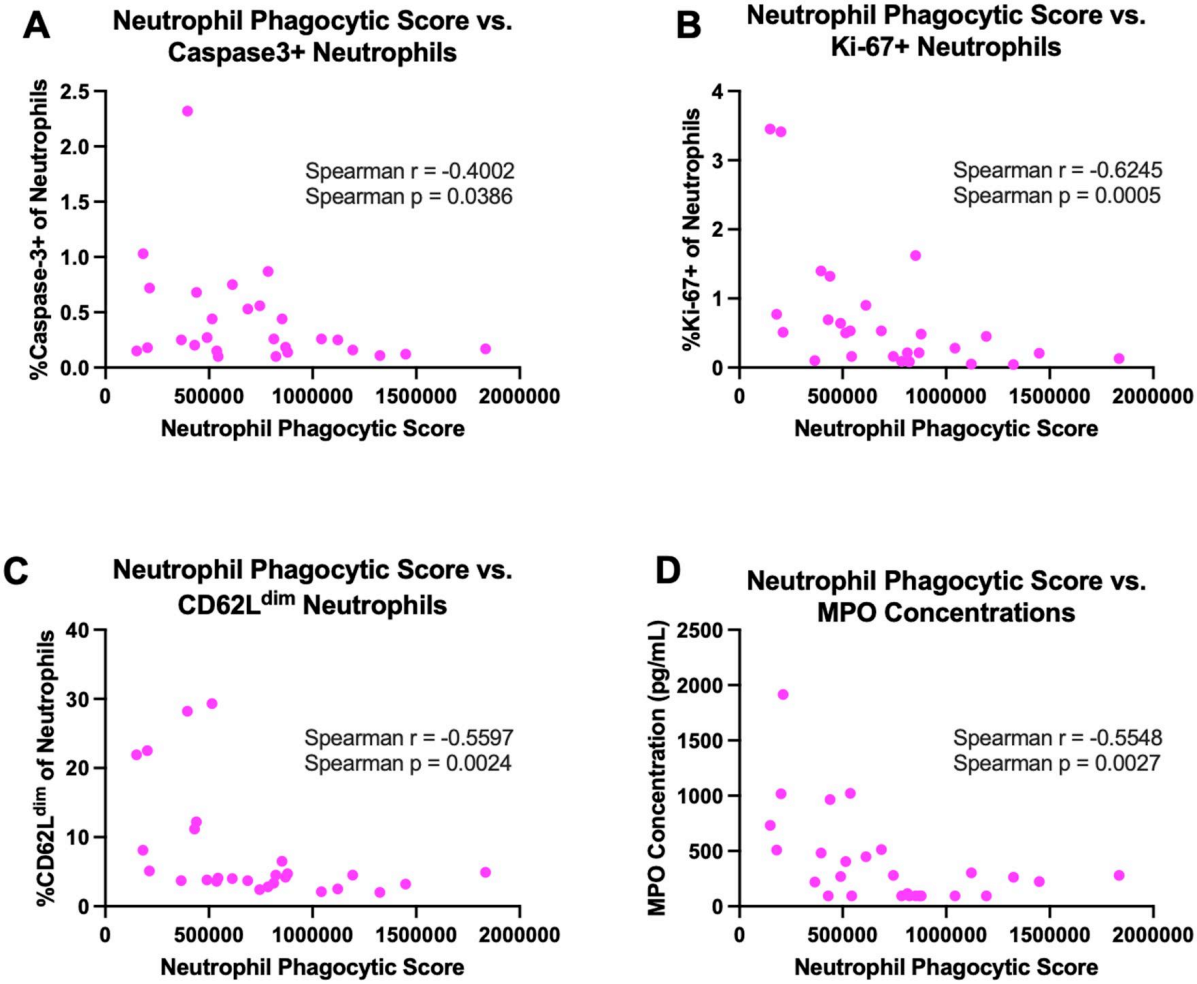
Correlations of peripheral neutrophil phagocytic capability with neutrophil phenotype and function during acute SIV and early ART initiation. Correlation of neutrophil phagocytic score with various parameters were computed via nonparametric Spearman correlation test. Correlations between neutrophil phagocytic score with (A) apoptotic, caspase3^+^ neutrophils, (B) Ki67^+^ neutrophils, (C) CD62L^dim^ neutrophils, and (D) peripheral concentrations of myeloperoxidase. Spearman’s rank *P* values and correlation coefficients (r) are indicated for each correlation.

To elucidate how phagocytic activity correlates with other aspects of neutrophil function, we next sought to examine the relationship between phagocytic score and peripheral neutrophil frequency and other markers of neutrophil function throughout infection and treatment. Neutrophil phagocytic score was inversely correlated with the frequency of apoptotic (caspase3^+^) neutrophils (Spearman’s rank correlation rho = −0.4002; *P* value = 0.0386; Fig. 8A). Phagocytic score of peripheral neutrophils was also inversely correlated with the frequency of Ki-67^+^ neutrophils (Spearman’s rank correlation rho = −0.6245; *P* value  = 0.0005; Fig. 8B), the frequency of CD62L^dim^ neutrophils (Spearman’s rank correlation rho = −0.5597; *P* value  = 0.0024; Fig. 8C), and MPO concentrations (Spearman’s rank correlation rho = −0.5548; *P* value  = 0.0027; Fig. 8D). Taken together, these findings suggest that neutrophil phagocytic capability is inversely associated with neutrophil apoptosis and indicators of neutrophil effector functions, such as NET formation, maturation and priming, and degranulation.

### Frequencies of activated CD4^+^ and CD8^+^ T cells are positively correlated with inflammatory neutrophil subsets, markers of NET formation, and neutrophil degranulation

**Figure 9.**
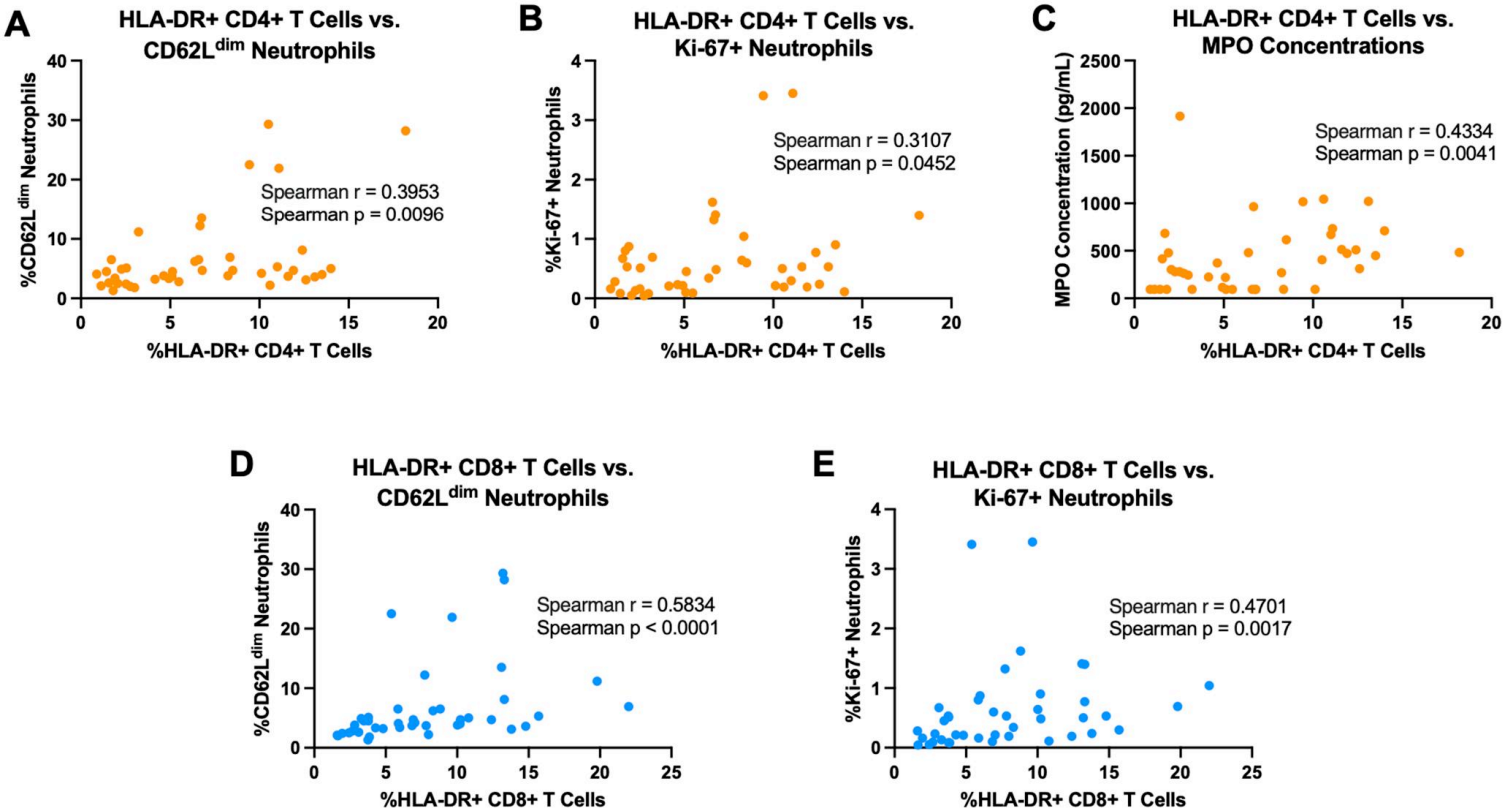
Activation of peripheral CD4^+^ and CD8^+^ T lymphocytes during acute SIV infection is correlated with inflammatory neutrophil phenotypes, biomarkers of NET formation, and neutrophil degranulation. Correlation of T cells with various parameters were computed via nonparametric Spearman correlation test. Correlations of CD4^+^ T cell activation are shown in orange; correlations include (A) CD62L^dim^ neutrophils, (B) Ki-67^+^ neutrophils, and (C) concentration of myeloperoxidase. Correlations of CD8^+^ T cell activation are shown in blue; correlations include (D) CD62L^dim^ neutrophils and (E) Ki-67^+^ neutrophils. Spearman’s rank *P* values and correlation coefficients (r) are indicated for each correlation.

Finally, to identify associations between neutrophils and activation and inflammation during acute SIV and following ART, we examined relationships between frequencies of activated CD4^+^ and CD8^+^ T cells and neutrophil subsets and markers of neutrophil function, including measurements from all experimental timepoints. The frequencies of HLA-DR^+^ CD4^+^ T cells were significantly positively correlated with CD62L^dim^ neutrophils (Spearman’s rank correlation rho = 0.3953; *P* value  = 0.0096; Fig. 9A), Ki-67^+^ neutrophils (Spearman’s rank correlation rho = 0.3107; *P* value  = 0.0452; Fig. 9B), and plasma concentrations of MPO (Spearman’s rank correlation rho = 0.4334; *P* value  = 0.0041; Fig. 9C). Likewise, the frequencies of HLA-DR+ CD8+ T cells were significantly positively correlated with Ki-67^+^ neutrophils (Spearman’s rank correlation rho = 0.4701; *P* value = 0.0017; Fig. 9D) and inflammatory CD62L^dim^ neutrophils (Spearman’s rank correlation rho = 0.5834; *P* value < 0.0001; Fig. 9E). Taken together, our data indicate that increased CD4^+^ and CD8^+^ T cell activation, hallmarks of pathogenic HIV/SIV infection, are positively associated with increases in markers of neutrophil effector function, including maturation and priming, NET formation, and degranulation.

## Discussion

Here, we characterized SIV viral dynamics and immune kinetics, including markers of systemic immune activation and neutrophil frequency, phenotype, and function during acute infection and following ART initiation in RMs. Our data on parameters of SIV infection in this cohort of female RMs, including viral load and T cell dynamics, align with what has previously been reported on acute SIV infection and ART treatment in RMs.^80^ Indeed, prior work has demonstrated that intravenous SIVmac239 inoculation results in elevated viral loads within 1 to 2 wk of infection,^81–84^ which is consistent with our finding here of peak viremia by week 2 p.i. We also observed depletion of peripheral CCR5^+^ CD4^+^ T cells during acute SIV infection and restoration to baseline levels with early ART initiation, which is consistent with prior reports on the CCR5-tropism of SIV,^85^^,^^86^ depletion of the CCR5^+^ CD4^+^ T cell compartment during acute SIV infection,^87^ and restoration of CCR5^+^ CD4^+^ T cells after ART.^88^ Peripheral CD8^+^ T cell proliferation was elevated during peak viremia in our cohort of RMs, but this was restored to baseline levels later in acute infection and sustained during ART, which is in line with previous reports that HIV-specific CD8^+^ T cells rapidly proliferate in acute infection but may thus become terminally differentiated effector cells and contribute to immune exhaustion.^47^^,^^48^^,^^89^^,^^90^ Moreover, we observed anemia and thrombocytopenia during acute SIV infection, which are previously reported hematological markers of HIV/SIV.^41–43^^,^^91^ In summary, the RMs included in our study followed expected parameters of acute SIV infection and ART treatment, thereby providing an opportunity to identify and examine potential innate immune contributors to immune activation, a strong predictor of HIV/SIV disease progression that persists even after initiation of suppressive therapy.

In this study, we did not observe major changes in hematological parameters of inflammation (SII, PNR, NMR, NLR). This could be attributed to early initiation of ART, which is clinically recommended and may have mitigated systemic inflammation as calculated by these ratios.^1^^,^^92^ Additionally, these hematological parameters of inflammation (SII, PNR, NMR, NLR) began to restore to baseline levels after acute infection and prior to ART initiation. Notably, however, the restored ratios were relatively stable after initiation of daily ART. Importantly, our assessment of these markers of systemic inflammation suggested that alterations in neutrophil dynamics occurred. Given previous studies suggesting a potential role for neutrophils in inflammation in the context of HIV/SIV,^30^^,^^93^ we therefore conducted a closer examination of peripheral neutrophil dynamics through SIV and following ART. We observed a significant decline in peripheral neutrophil frequencies during acute SIV infection prior to ART initiation, which is in agreement with previous reports of neutropenia in PWH,^94^^,^^95^ as well as during acute SIV infection in RMs.^96^ Indeed, a study by Hensley-McBain et al. demonstrated significantly decreased neutrophil frequencies at week 9 p.i. in peripheral blood of RMs intrarectally inoculated with SIVmac239X.^57^ A potential reason that we observed an earlier significant decline of peripheral neutrophils in this cohort of RMs (week 2 p.i.) compared to the study by Hensley-McBain et al. is that we used intravenous inoculation of SIV, compared to intrarectal inoculation. Moreover, ART treatment in PWH has been shown to restore neutropenia^71^ and neutrophil function.^95^^,^^97^ Additionally, peripheral neutrophil apoptosis was shown to be increased in RMs chronically infected with SIVmac251, particularly in animals that rapidly progressed to AIDS.^59^ Our observation that frequencies of total and apoptotic neutrophils were restored to baseline levels following two months of ART in SIV-infected RMs are therefore in line with this prior work. Of note, while we observed a decline in neutrophil frequencies during acute SIV infection in this cohort of RMs, we are unable to delineate whether this is attributed to neutrophil depletion or trafficking of neutrophils into tissues. Taken together, our data and these previous findings suggest that SIV significantly alters peripheral neutrophil dynamics, which may resolve after acute infection and remain stable throughout ART.

During peak viremia in our cohort of animals, we observed a reduction in neutrophil phagocytic ability coupled with increased biomarkers of neutrophil degranulation (MPO) and neutrophil expression of Ki-67, which may indicate a prevalence of neutrophils with a propensity for NET formation. These changes began to restore to baseline levels later in acute infection and were maintained to these levels after ART initiation. Prior work has suggested that neutrophils undergo a dichotomous choice between phagocytosis and NET formation.^98^^,^^99^ Neutrophil phagocytosis of apoptotic cells and cellular debris initiates the resolution of inflammation,^100^ while degranulation and NET formation may exacerbate inflammatory responses.^101^ Notably, in this cohort of RMs, we observed a reduction in neutrophil phagocytic capability at peak viremia. This is consistent with prior reports of impaired neutrophil phagocytosis in PWH,^15^^,^^102^^,^^103^ but to the best of our knowledge, this is the first observation of this phenomenon in SIV-infected RMs. It is reasonable to speculate that circulating neutrophils unable to phagocytose underwent the decisive shift toward degranulation and NET formation, suggested by our observations of increased levels of MPO and the frequency of Ki-67 expressing neutrophils. Importantly, we observed a significant increase in neutrophil phagocytic ability after 1 mo of ART, which was coupled with a decrease in levels of MPO and frequency of Ki-67+ neutrophils, further supporting a potential inverse relationship between neutrophil phagocytic activity and neutrophil degranulation/NET formation in the context of acute SIV infection and ART.

Elevated levels of NETosis during HIV infection have been shown to be decreased in the context of ART.^30^ These prior findings are consistent with our data indicating that an increase in Ki-67^+^ neutrophils returned to baseline levels following ART-initiation. However, we did not observe changes in NE or CitH3, which have been used as peripheral biomarkers of NET formation.^72^^,^^73^^,^^104–110^ This finding is interesting in light of the significant increase of Ki-67^+^ neutrophils at peak viremia. Amulic et al. demonstrated that NETosis is accompanied by Ki-67 upregulation in neutrophils, and this upregulation of Ki-67 temporally precedes NET formation. A possible explanation for our findings here is that neutrophils observed in the periphery upregulated Ki-67 expression prior to trafficking to tissues to form NETs. Given that excessive NET formation may contribute to destruction of immune cells, inflammation, and tissue damage,^111^^,^^112^ our data further support the importance of early ART initiation to limit NET formation as a means to limit a potential mechanism of uncontrolled inflammation. However, more work will be needed to identify the precise contributions of Ki-67^+^ neutrophils to NET formation both in the periphery and pertinent tissue sites in the context of acute SIV infection and ART. Additionally, a limitation of the data presented here is that surrogate markers, namely, NE and CitH3, were used to assess peripheral NET formation.^72–75^^,^^104–110^ Quantification of these molecules may not capture the dynamic complexity of NETosis that occurs during SIV infection, which may further help to explain the discrepancy we observed between expression of these biomarkers and the indication of potential NET formation via Ki-67 expression by neutrophils. Future work with functional assays to directly evaluate NETosis in the context of ART-treated SIV infection are warranted.

A key finding of our study is our observation that changes in neutrophil frequency, phenotype, and function correlated with increased markers of T cell activation. A growing body of literature suggests that neutrophils play a role in the coordination of adaptive immune responses.^113^^,^^114^ Moreover, prior work has suggested that neutrophil phenotypes may be impacted by CD4^+^ T cell levels in PWH, and that there may be a potential link between T cell activation and neutrophil profiles during HIV infection.^115^ Although not significant, our immunophenotypic analysis of circulating neutrophils revealed a transient increase in CD62L^dim^ neutrophils, consistent with previous reports of expanded populations of CD62L^dim^ neutrophils during acute SIV infection.^62^ This subset of primed CD62L^dim^ neutrophils is recruited to the bloodstream during acute inflammatory conditions and engages in immune regulation through cytokine production.^61^^,^^116^ Lemaitre et al. observed progressive reduction in this CD62L^dim^ neutrophil subset after initiation of ART 1 mo post-SIV inoculation,^62^ which is consistent with our observations in this cohort of RMs. While significant longitudinal changes in CD62L^dim^ neutrophils were not evident in our cohort, we did observe significant correlations of this neutrophil subset with markers of immune activation. Taken together, these prior findings in conjunction with our data suggest a potential association between inflammatory neutrophil responses and immune activation during SIV infection, and that ART initiation may help to limit the frequency of inflammatory neutrophils and level of T cell activation during HIV/SIV. Future mechanistic studies to evaluate the role of neutrophils in driving systemic immune activation and enhancing the risk for inflammation-associated adverse outcomes during HIV/SIV infection are warranted.

A major strength of our study was utilization of the NHP model, which demonstrates anatomical and immunological similarities to humans.^117^ Specifically, intravenous SIVmac239 inoculation of this cohort of female RMs resulted in a disease profile similar to that previously reported in PWH, characterized by peripheral viral dynamics, CCR5^+^ CD4^+^ T cell depletion, and peripheral immune responses.^15^^,^^118^^,^^119^ Our experimental design enabled longitudinal assessment of peripheral neutrophil dynamics in the context of acute SIV infection and ART. Moreover, our experimental timeline is clinically relevant, as current treatment guidelines recommend that PWH initiate ART as soon as possible after diagnosis, as early ART initiation reduces risk of mortality and AIDS progression.^1^^,^^92^^,^^120^

A caveat of our study is the relatively small number of animals used (n = 6 females), particularly given the fact that we observed inter-animal variation in certain parameters. This may have contributed to the lack of statistical significance observed at some time points. Furthermore, we only monitored animals up to week 20 p.i. with 2 mo of daily ART, so we were unable to draw conclusions about the impact of long-term ART on neutrophil dynamics and function in SIV-infected RMs. Additionally, because we initiated ART early in acute infection, we were unable to examine the development of chronic immune activation in this cohort of animals. Additional work with larger animal cohorts and extended experimental timelines will be required to comprehensively evaluate the impact of ART-treated SIV infection on neutrophil dynamics and function. Specifically, future work could evaluate whether decreases of neutrophil frequencies during acute SIV are due to neutrophil depletion or neutrophil trafficking to relevant tissue compartments, including the gastrointestinal and female reproductive tracts.

In conclusion, our data demonstrate that SIVmac239 infection of female RMs results in clinical signs of acute SIV infection, including high VL and depleted CCR5^+^ CD4^+^ T cells, which resolve following 2 months of ART. Moreover, we observed significant alterations in peripheral neutrophil frequencies, phenotype, and function during acute SIV and ART that correlated with frequencies of activated T lymphocytes, potentially suggesting a role for neutrophils in driving inflammation and immune activation during SIV infection that may be alleviated with ART. Further characterization of the mechanism underlying this association is critical for elucidating the interplay between neutrophils, systemic inflammation, and immune activation during SIV infection and will open the possibility of identifying potential immune-based therapies that can be used in conjunction with ART to reduce the risk of inflammation-associated comorbidities in PWH.

## Supplementary Material

vkaf100_Supplementary_Data

## Data Availability

All data that support the findings of this study are available from the corresponding author upon reasonable request.
