# Supplementary material for: Inflammatory neutrophil responses and T cell activation in ART-treated SIVmac239-infected rhesus macaques
Source: J Immunol. 2025 Jun 12;214(9):2307–24. doi: 10.1093/jimmun/vkaf100 (PMC12481039; doi:10.1093/jimmun/vkaf100)
Supplement: vkaf100_Supplementary_Data [file vkaf100_supplementary_data.pdf]

Supplemental Table 1

Supplemental Table 1. Staining reagents utilized for identification, immunophenotyping, evaluation of phagocytosis, and absolute count quantification of immune cell subsets in whole blood. Color, marker name, clone, company, catalog number, host, and staining step of antibodies used in flow cytometric staining.

Immunophenotype Flow Cytometry Panel						
**Color**	**Marker**	**Clone**	**Company**	**Catalog #**	**Host**	**Staining Step**
FITC	NKp44	2.29	Miltenyi	130-118-542	Mouse IgG1, κ	Extracellular
BB630	CD69	FN50	BD Biosciences	Custom	Mouse IgG1, κ	Extracellular
BB660	CD45	D058-1283	BD Biosciences	558411	Mouse IgG1, κ	Extracellular
BB700	CCR5	3A9	BD Biosciences	746211	Mouse IgG2a, κ	Chemokine
BB750	CD62L	Sk11	BD Biosciences	Custom	Mouse IgG2a, κ	Extracellular
BB790	Ki-67	B56	BD Biosciences	Custom	Mouse IgG1, κ	Intracellular
BV421	CD66abce	TET2	Miltenyi	130-119-851	Mouse IgG2b, κ	Extracellular
BV510	CD3e	SP34-2	BD Biosciences	740187	Mouse IgG1, λ	Extracellular
BV570	CD16	3G8	BioLegend	302036	Mouse IgG1, κ	Extracellular
BV605	CD86	2331 (FUN-1)	BD Biosciences	562999	Mouse IgG1, κ	Extracellular
BV650	CD11b	ICRF44	BioLegend	301336	Mouse IgG1, κ	Extracellular
BV711	c-Kit (CD117)	104D2	BioLegend	313230	Mouse IgG1, κ	Extracellular
BV750	CD8	SK1	BD Biosciences	747097	Mouse IgG1, κ	Extracellular
BV785	CD68	Y1/82A	BioLegend	333826	Mouse IgG2b, κ	Intracellular
BUV395	CD11c	SHCL-3	BD Biosciences	744440	Mouse IgG2b	Extracellular
DAPI	Live/Dead UV	N/A	BioLegend	423108	N/A	Live/Dead
BUV496	HLA-DR	G46-6	BD Biosciences	749866	Mouse IgG2a, κ	Extracellular
BUV563	CD163	GHI/61	BD Biosciences	741402	Mouse IgG1, κ	Extracellular
BUV615	CD56	B159	BD Biosciences	751349	Mouse IgG1, κ	Extracellular
BUV661	CD20	2H7	BD Biosciences	749952	Mouse IgG2b, κ	Extracellular
BUV737	CD123	7G3	BD Biosciences	741769	Mouse IgG2a, κ	Extracellular
BUV805	CD4	OKT4	BD Biosciences	750976	Mouse IgG2b, κ	Extracellular
PE	ST2/IL-33R	Polyclonal	R&D Systems	FAB5231P	Goat IgG	Extracellular
PE/Dazzle594	CD169	7-239	BioLegend	346016	Mouse IgG1, κ	Extracellular
PE-Cy5	CD14	M5E2	BioLegend	301864	Mouse IgG2a, κ	Extracellular
PE-Cy5.5	CD127	eBioRDR5	Invitrogen	35-1278-42	Mouse IgG1, κ	Extracellular
PE-Cy7	NKG2A/C	Z199	BeckmanCoulter	B10246	Mouse IgG2b	Extracellular
AL647	Caspase3	C92-605	BD Biosciences	560626	Rabbit IgG	Intracellular
R718	CD206	19.2	BD Biosciences	751998	Mouse IgG1, κ	Intracellular
APC-A750	CD49d	HP2/1	BeckmanCoulter	B16893	Mouse IgG1	Extracellular
**Neutrophil Phagocytosis Flow Cytometry Panel**						
**Color**	**Marker**	**Clone**	**Company**	**Catalog #**	**Host**	**Staining Step**
FITC	CD3	SP34	BD Biosciences	556611	Mouse IgG3, λ	Extracellular
FITC	CD20	2H7	BioLegend	302304	Mouse IgG2b, κ	Extracellular
PerCP	CD66abce	TET2	Miltenyi	130-119-850	Mouse IgG2b, κ	Extracellular
APC	CD49d	HP2/1	Beckman Coulter	B01682	Mouse IgG1	Extracellular
APC_Cy7	CD11b	ICRF44	BD Biosciences	557754	Mouse IgG1, κ	Extracellular
BV.510	Live/Dead	N/A	Invitrogen	L34965	N/A	Live/Dead
BV.650	CD16	3G8	BD Biosciences	563691	Mouse IgG1, κ	Extracellular
BV.786	HLA-DR	G46-6	BD Biosciences	564041	Mouse IgG2a, κ	Extracellular
PE_Cy5	CD14	M5E2	BioLegend	301864	Mouse IgG2a, κ	Extracellular
PE_Cy7	CD11c	S-HCL-3	BioLegend	371508	Mouse IgG2b, κ	Extracellular
BUV.395	CD45	D058-1283	BD Biosciences	564099	Mouse IgG1, κ	Extracellular
BUV.737	CD123	7G3	BD Biosciences	741769	Mouse IgG2a, κ	Extracellular
**CD4+/CD8+ T Cell Absolute Count Flow Cytometry Panel**						
**Color**	**Marker**	**Clone**	**Company**	**Catalog #**	**Host**	**Staining Step**
FITC	CD3	SP34	BD Biosciences	556611	Mouse IgG3, λ	Extracellular
PerCP	CD45	D058-1283	BD Biosciences	558411	Mouse IgG1, κ	Extracellular
APC	CD4	L200	BD Biosciences	551980	Mouse IgG1, κ	Extracellular
V500	CD8	SK1	BD Biosciences	561618	Mouse IgG1, κ	Extracellular

Supplemental Figure 1

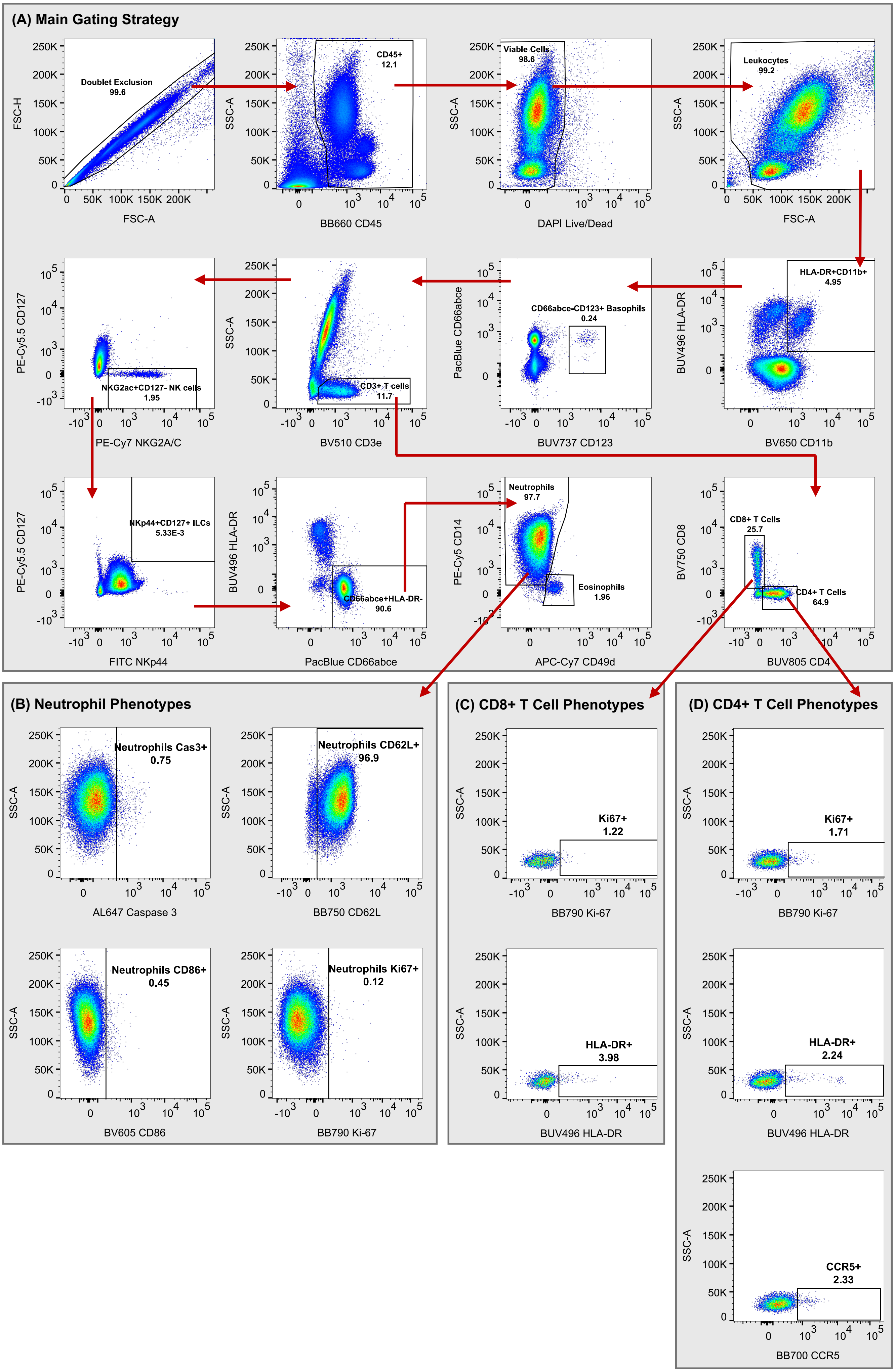

Supplemental Figure 1

Supplemental Figure 1. Representative flow cytometry gating strategy for identification of immune cells in rhesus macaque whole blood. Depicted here are representative plots of stained whole blood from rhesus macaque (RM23-0172) at baseline timepoint prior to SIVmac239 inoculation (week -10). (A) In the main gating strategy, cells were identified first by excluding doublets using forward scatter area (FSC-A) and forward scatter height (FSC-H) properties, gating on CD45+ cells, excluding dead cells using Fixable Blue Live/Dead viability dye, and removing any remaining debris was excluded using FSC-A and side scatter area (SSC-A) properties. Next, HLA-DR+CD11b+ cells (B cells, macrophages, monocytes, dendritic cells) were excluded using a NOT Boolean gate. Basophils were identified as CD66abce-CD123+. After excluding basophils using a NOT Boolean gate, T cells were identified as CD3+, and CD4+ and CD8+ T cells were gated within CD3+ cells. After excluding CD3+ T cells with a NOT Boolean gate, natural killer cells were identified as NKG2AC+CD127-. Exclusion of NK cells with a NOT Boolean gate enabled identification of innate lymphoid cells as NKp44+CD127+. After excluding ILCs using a NOT Boolean gate, CD66abce+HLA-DR- identified neutrophils, CD14+CD49d-,and eosinophils, CD14^{dim}CD49d+. (B) Frequencies of neutrophils expressing caspase 3, CD62L, CD86, and Ki-67 were defined as a percent of total neutrophils. (C) Frequencies of CD8+ T cells expressing Ki-67 and HLA-DR were defined as a percent of total CD8+ T cells. (D) Frequencies of CD4+ T cells expressing Ki-67, HLA-DR, and CCR5 were defined as a percent of total CD4+ T cells.

Supplemental Figure 2

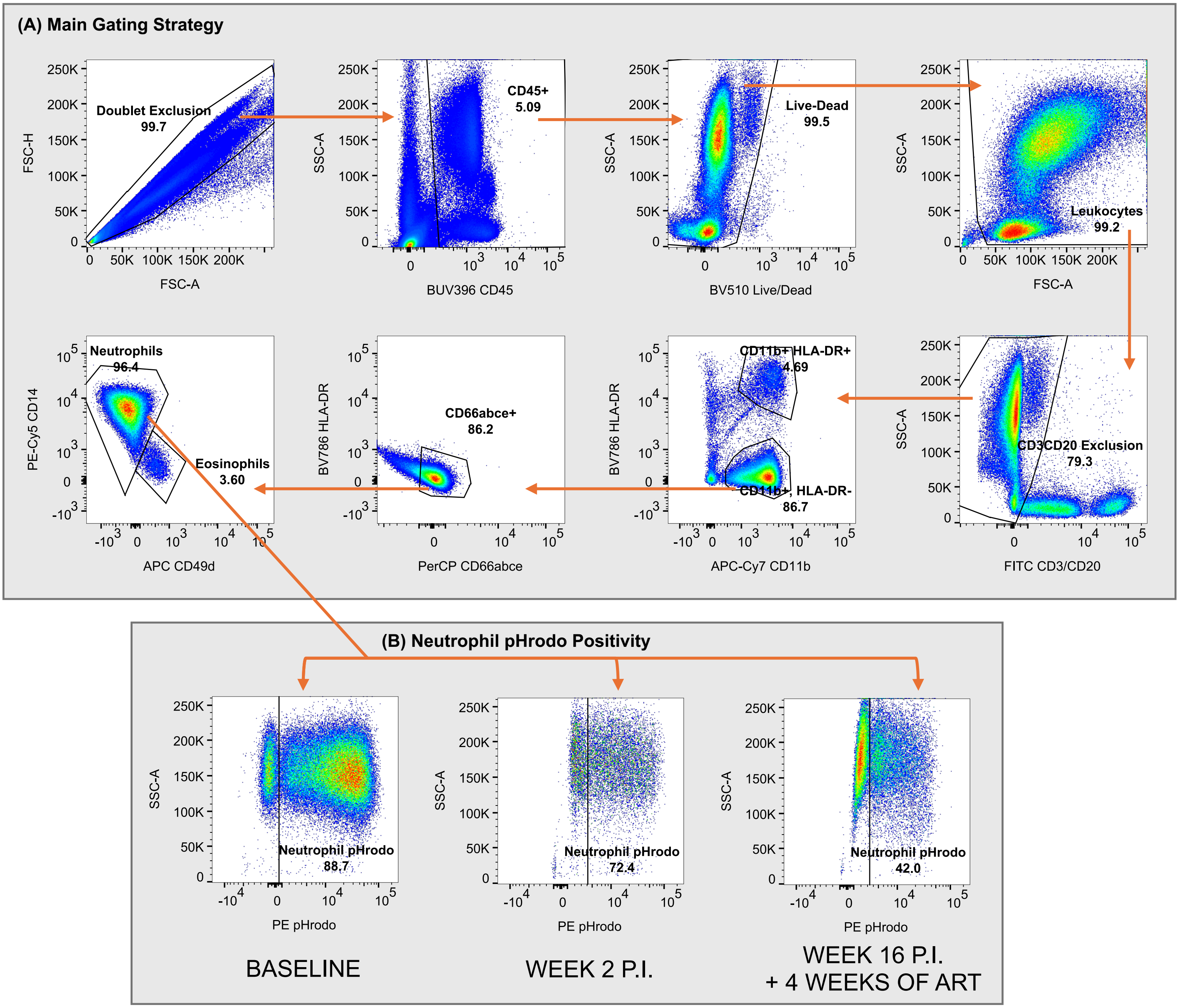

Supplemental Figure 2. Representative flow plots demonstrating gating strategy to evaluate neutrophil phagocytosis via uptake of pHrodo bioparticles. (A) Representative gating strategy plots depict data from whole blood from rhesus macaque (RM23-0172) at baseline timepoint prior to SIVmac239 inoculation (week -2). Representative neutrophil pHrodo bioparticle positivity taken from timepoints week -2, week 2 p.i., and week 16 p.i.. Doublets were excluded using forward scatter area (FSC-A) and forward scatter height (FSC-H) properties. Singlet CD45+ cells were identified, and dead cells were excluded using Aqua Live/Dead viability dye. Remaining debris was excluded using FSC-A and side scatter area (SSC-A) properties. Next, lymphocytes (CD3+ and CD20+) were excluded using a NOT Boolean gate. Next, granulocytes were identified as CD11b+ HLA-DR- cells. Among granulocytes, neutrophils and eosinophils were identified as CD66abce+ cells. Of these CD66abce+ cells, neutrophils were identified as CD14+ CD49d-, and eosinophils were identified as CD14^{dim} CD49d+. (B) Percent of phagocytosing neutrophils was defined as pHrodo+ neutrophils among total neutrophils.
